# Toward identification of a putative candidate gene for nutrient mineral accumulation in wheat grains for human nutrition purposes

**DOI:** 10.1093/jxb/erab297

**Published:** 2021-06-18

**Authors:** Dalia Z Alomari, Ahmad M Alqudah, Klaus Pillen, Nicolaus von Wirén, Marion S Röder

**Affiliations:** 1 Leibniz Institute of Plant Genetics and Crop Plant Research (IPK), Corrensstrasse 3, D-06466 Stadt Seeland OT Gatersleben, Germany; 2 Institute of Agricultural and Nutritional Sciences, Martin-Luther-University Halle-Wittenberg, Betty-Heimann-Str. 3, 06120 Halle/Saale, Germany; 3 CSIRO Agriculture and Food, Australia

**Keywords:** Grain minerals, GWAS, Major Facilitator Superfamily transporter, ICP-OES, *Triticum aestivum*, wheat

## Abstract

A multilocus genome-wide association study of a panel of 369 diverse wheat (*Triticum aestivum*) genotypes was carried out in order to examine the genetic basis of variations in nutrient mineral concentrations in the grains. The panel was grown under field conditions for three consecutive years and the concentrations of Ca, K, Mg, Mn, P, and S were determined. Wide ranges of natural variation were detected among the genotypes. Strong positive correlations were found among the minerals except for K, which showed negative correlation trends with the other minerals. Genetic association analysis detected 86 significant marker–trait associations (MTAs) underlying the natural variations in mineral concentrations in grains. The major MTA was detected on the long arm of chromosome 5A and showed a pleiotropic effect on Ca, K, Mg, Mn, and S. Further significant MTAs were distributed among the whole genome except for chromosomes 3D and 6D. We identified putative candidate genes that are potentially involved in metal uptake, transport, and assimilation, including *TraesCS5A02G542600* on chromosome 5A, which was annotated as a Major Facilitator Superfamily transporter and acted on all the minerals except K. *TraesCS5A02G542600* was highly expressed in seed coat, and to a lesser extent in the peduncle, awns, and lemma. Our results provide important insights into the genetic basis of enhancement of nutrient mineral concentrations that can help to inform future breeding studies in order to improve human nutrition.

## Teaser

A multilocus GWAS for a diverse wheat panel highlights several putative candidate genes for mineral accumulation in the grains, with a Major Facilitator Superfamily transporter showing the most significant marker–trait association in enhancing the nutritional value.

## Introduction

Cereal-based foods represent the largest proportion of the human daily diet consumed worldwide and wheat (*Triticum aestivum*) is the primary protein source in developing countries, with 2.5 billion consumers in 89 countries (https://wheat.org/). However, wheat grain is inherently relatively low in nutrients, where ‘nutrients’ refers to a broad range of minerals and vitamins that play important roles in the biological functioning of the human body (Tapiero *et al.*, 2003; Pujar *et al.*, 2020).

Many wheat research programs have focused on increasing yields whilst ignoring grain quality, leading to depletion in the nutrient composition and decreased nutritional value. This can lead to high incidences of nutrient deficiencies and malnutrition occurring in countries that rely on a cereal-based diet and where bread wheat forms the majority of the daily intake of calories. For example, the World Health Organization has reported that in 2012, 162 and 99 million children were stunted and underweight, respectively, mainly because of insufficient intake of essential nutrients (WHO, 2013). The importance of developing wheat genotypes with improved nutrient contents in the grains is therefore clear.

Wheat is the second most important staple crop worldwide: it is cultivated on almost 200×10^6^ ha, feeds more than a third of the world’s population, and provides a fifth of the total calories consumed worldwide (Godfray *et al.*, 2010). Ray *et al.* (2013) anticipated that by 2050 the food demand for wheat in particular would increase by 35–40%. In response, recent research has begun to focus on exploiting natural variations among genotypes to identify those that have not only good yields but also high quality in terms of nutritional value (Alomari *et al.*, 2017; Yu and Tian, 2018).

Recent developments in molecular markers and genome sequencing technologies combined with the release of the high-quality wheat reference genome sequence allow plant researchers to characterize the genetic basis of complex phenotypic traits by using hundreds of thousands of genetic markers in association mapping and in the detection of quantitative trait loci (QTLs) (Appels *et al.*, 2018). Applying high-density marker arrays for single-nucleotide polymorphisms (SNPs) combined with a suitable approach such as genome-wide association studies (GWAS) can identify robust QTLs and help detect genes underlying complex phenotypic traits (Rasheed and Xia, 2019; Alqudah *et al.*, 2020). Nutrient accumulation in wheat grains is one such example, as it is a function of complex inherited traits that are controlled by several different factors, including nutrient uptake by the roots from the soil, translocation, assimilation, and remobilization to the grains (Sperotto *et al.*, 2014).

Another approach that has recently become popular in plant breeding research is based on using molecular markers covering the whole genome, the so-called genomic prediction (GP) approach. This uses genome-wide marker information to predict the breeding value of complex traits in order to accelerate breeding programs (Meuwissen *et al.*, 2001). The accuracy of GP can vary according to the prediction method that is used, as different assumptions and treatments of marker effects and models are utilised (Desta and Ortiz, 2014).

In the current study, we aimed to examine the natural variations in nutrient mineral accumulation in grains of elite European hexaploid winter wheat genotypes. Using high-density SNP arrays, we attempted to detect stable genomic regions associated with natural variations in calcium (Ca), magnesium (Mg), manganese (Mn), phosphorus (P), potassium (K), and sulphur (S) based on multi-locus GWAS, and to assess their value in terms of genomic prediction and breeding potential. The ultimate goal was to identify the most relevant candidate genes potentially involved in controlling mineral accumulation in wheat grains with a view to assisting future breeding programs aimed at crop improvement.

## Materials and methods

### Plant material

The wheat (*Triticum aestivum*) germplasm that we used consisted of 369 European elite registered varieties, made up of 355 winter varieties and 14 spring varieties (Supplementary Table S1). The genotypes were mostly from Germany and France. Field experiments were conducted at IPK, Gatersleben, Germany (51°490´N, 11°170´E), during the growing seasons of 2014/2015, 2015/2016, and 2016/2017, with the full set of genotypes being grown each year in plots of 2×2 m with six rows spaced 0.20 m apart. All varieties were subjected to standard agronomic wheat management practices.

### Grain sampling and measurement of nutrients

For each genotype, ears were harvested by hand from the whole plot and a randomly selected sample of kernels were placed in a MARViN digital seed analyser/counter (GTA Sensorik GmbH, Neubrandenburg, Germany) and 50 were collected for mineral analysis. These kernels were also used for determination of thousand-kernel weight (TKW). The samples for mineral analysis were ground using a Mixer Mill MM 300 (Retsch GmbH, Germany) and dried at 40 °C in an incubator overnight. The concentrations of Ca, K, Mg, Mn, P, and S were measured by inductively coupled plasma optical emission spectrometry (ICP-OES) using an iCAP 6000 (ThermoFisher Scientific) combined with an ASXPRESS PLUS Rapid Sample Introduction system and an ASX-560 autosampler (both Teledyne CETAC Technologies, Omaha, NE, USA). Each ground sample was subjected to wet digestion in 2 ml 69% nitric acid (HNO_3_) using a high-performance microwave reactor (UltraClave IV, MLS, Germany). The digested samples were made up to 15 ml final volume with de-ionized distilled water. Element standards were prepared from Bernd Kraft GmbH multi-element standard solution (Duisburg, Germany). Standard solutions were used for determination of the minerals, and yttrium was used as an internal standard for matrix correction (ICP Standard Certipur, Merck, Germany).

### Genotyping and quality control for markers

The wheat panel was genotyped using two marker arrays: a 90K iSELECT Infinium array including 7761 markers and a 35K Affymetrix SNP array including 7762 markers (Wang *et al.*, 2014) (Axiom^®^ Wheat Breeder’s Genotyping Array, https://www.cerealsdb.uk.net/cerealgenomics/). Both were genotyped by SGS-TraitGenetics GmbH, Gatersleben, Germany (www.traitgenetics.com) as detailed previously (Alomari *et al.*, 2019). The ITMI-DH population was used as a reference map to anchor the SNP markers of the two arrays (Sorrells *et al.*, 2011; Poland *et al.*, 2012). To obtain high-quality makers, the SNP markers of the 90K and 35K chips were subjected to a quality control and filtration process by removing those with ≤3% missing values, a minor allele frequency (MAF) of ≤3%, and those with unknown chromosomal positions. We then used the physical position of wheat genome sequence RefSeq v.1.0 for the SNPs.

### GWAS and genomic prediction

The GWAS analysis was carried out using the Genomic Association and Prediction Integrated Tool (GAPIT) in the R software (Lipka *et al.*, 2012). First, the GWAS analysis was computed using a mixed linear model (MLM) that took into account the variance–covariance kinship matrix and principle component analysis (PCA), and conducted by incorporating the phenotypic and genotypic dataset. The kinship matrix was calculated using the VanRaden method (VanRaden, 2008) to determine relative kinship among the sampled individuals. Both PCA and kinship matrix were used for population correction and stratification.

We also applied a GAPIT model known as ‘fixed and random model circulating probability unification’ (FarmCPU) to our data analysis. This was applied by considering the incorporation of multiple markers simultaneously as a covariate in a fixed-effects model and optimization on the associated covariate markers separately in a random effects model, which enabled us to avoid any false-negatives and to control the false-positive associations by preventing model over-fitting (Liu *et al.*, 2016). Thus, FarmCPU is a powerful tool with less false-positives than MLM. The selection of an appropriate model and thresholds are important steps in identifying markers that are truly associated with specific traits and that might be located within or very close to genes that control the trait variation while also controlling both false-positive and false-negative associations. To determine which of the tested models best fitted the data, we plotted quantile-quantile (Q-Q) plots based on the observed and expected –log_10_(*P*) values. Based on the GWAS output of three models (GLM, MLM, and FarmCPU), the number of significant associations, and the resulting Q-Q plots, we then selected the FarmCPU model. A threshold *P*-value 0.001 equal to –log_10_(*P*)≥3 was used to determine the significance of marker–trait associations, and then Bonferroni correction at *P*<0.05 was used to adjust the –log_10_(*P*) threshold to a value of 5.49 for the studied traits.

Marker effects (positive or negative) and phenotypic variance explained by the associated markers (*R*^2^) were removed from the GWAS results.

Ridge regression–best linear unbiased prediction (RR-BLUP) and Bayes-Cπ methods were used to evaluate the genomic predictions (Meuwissen *et al.*, 2001; Habier *et al.*, 2011), with both methods being implemented in the R software. Five-fold cross-validation was applied to the complete set, with the set being randomly divided into five subsets, four of which were used as estimation sets and the remaining ones were used as the test set. The prediction ability was calculated from the correlation between the observed and predicted values. The whole process was repeated 100 times to obtain a mean value. 

### Gene identification, annotation, and expression analysis

Significant markers and the markers located within the linkage disequilibrium region (*r*^2^≥0.2) were considered for BLAST. The sequences of the identified makers were obtained from the wheat 90K (Wang *et al.*, 2014) and 35K databases (Allen *et al.*, 2017). Marker sequences were BLASTed against the recently released IWGSC RefSeq v.1.1 genome using EnsemblPlants (http://plants.ensembl.org/Triticum_aestivum) to obtain their gene annotations. The expression profiles of all the putative candidate genes associated with the identified SNPs obtained from the published RNA-seq expression database for wheat in the WheatGmap web tool (https://www.wheatgmap.org; Zhang *et al.*, 2020).

### Statistical analysis

Significant differences in minerals among genotypes and years were determined using ANOVA and Pearson correlation coefficients were determined to evaluate strengths of relationships among the measured parameters, both using GenStat v.19. This software was also used to determine best linear unbiased estimates (BLUEs) over the three years of the experiments by restricted maximum likelihood (REML) analysis with a mixed linear model (MLM) where the genotype considered as a fixed effect and the environment as a random effect. BLUEs were calculated for each genotype of each trait across the years.

Broad-sense heritability was calculated for each trait using the formula *H*^2^=*V*_G_/[*V*_G_+(*G*_e_/*nE*)], where *V*_G_ is the variance of the genotype, *G*_e_ is the variance of the residual, and *nE* is the number of years.

## Results

### Genetic variation and correlations

**Fig. 1.**
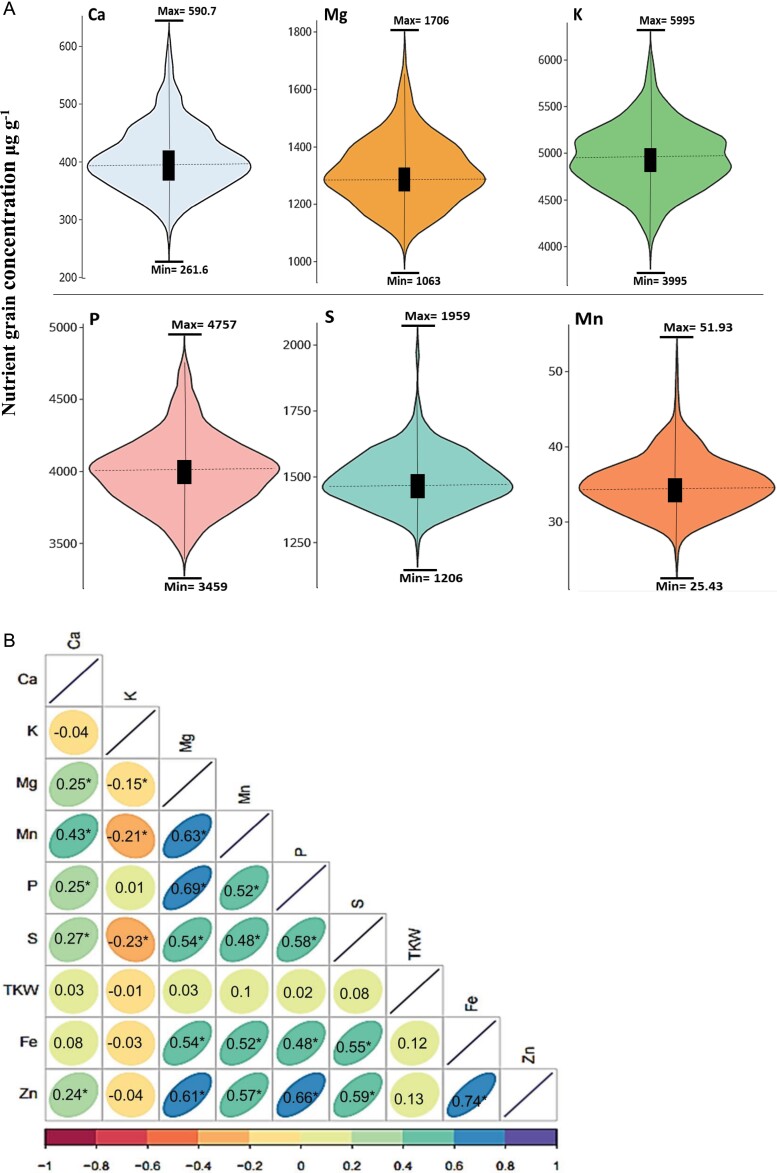
Mineral nutrient concentrations in the grains of 369 European wheat genotypes grown in over three seasons. (A) Violin plots mineral concentrations. (B) Pearson correlations among mineral concentrations and thousand-kernel weight (TKW) based on best linear unbiased estimates (BLUEs). Significant correlations were determined using ANOVA (*P≤0.05).

The wheat genotypes showed wide variations in mineral concentrations in the grains across the three years (Fig. 1a) and BLUEs that followed approximately normal distributions (Supplementary Fig. S1). The variation ranged from 208–797 μg g^–1^ for Ca, 3495–6727 μg g^–1^ for K, 963–1988 μg g^–1^ for Mg, 23.3–62.2 μg g^–1^ for Mn, 2943 to 5807 μg g^–1^ for P, and 974–2368 μg g^–1^ for S (Supplementary Table S2). Concentrations of Ca and S were highest in 2015, K, P, and Mn concentrations were highest in 2016, and Mg concentration did vary appreciably over the three years. This variation among years could be explained by environmental influences (Supplementary Table S3). Correlation analysis was performed for the nutrient traits and for thousand-kernel weight (TKW) based on BLUE values (Fig. 1B). Strong positive correlations were found between Mg and P, and between Mg and Mn (*r*=0.69 and *r*=0.63, respectively, *P*<0.05), while moderate positive correlations were detected between Ca and Mg (*r*=0.25), Mn (*r*=0.43), P (*r*=0.25), and S (*r*=0.270 (all *P*<0.01). Negative correlations were detected between K and Ca, Mg, Mn, and S, but this was not significant for both Ca. All the minerals showed only very weak correlations with TKW. . In this study, the five genotypes with highest measured mineral concentrations were Isengrain, Inoui, Nirvana, Exotic, and Lona.

ANOVA indicated that significant differences concentrations existed among the genotypes (*P*<0.001) and the years (*P*<0.001) for all the minerals, except Mn where there was no effect of year (Supplementary Table S3). The broad-sense heritability (*H*^*2*^) values were high and ranged from 0.72 for Mn to 0.87 for Ca. These results indicated that there was considerable natural variation in mineral concentrations that were predominantly genetically controlled with a relatively low influence of the environment. This suggested that it might be possible to detect shared genomic regions for the mineral traits.

### Association analysis and genomic predictions

**Fig. 2.**
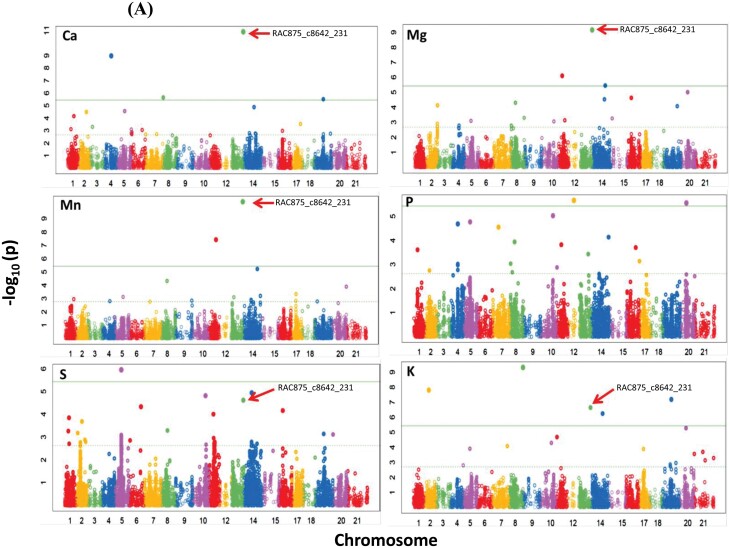

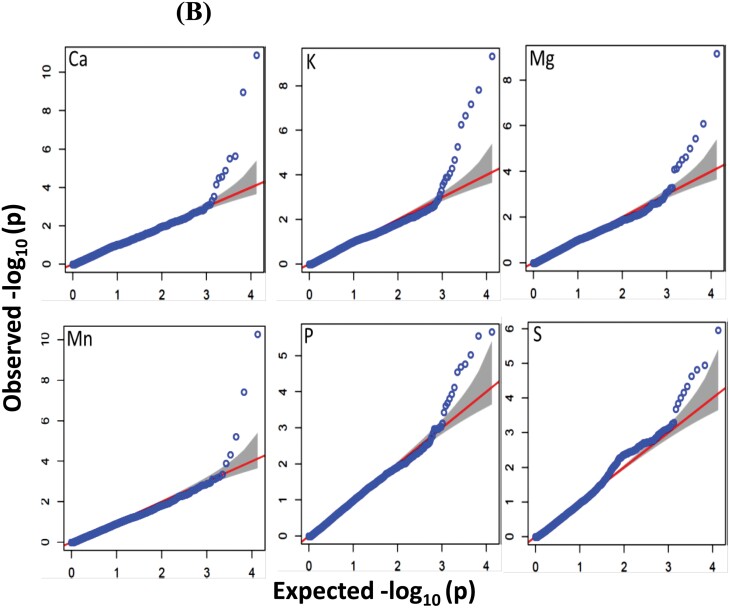
Genome-wide association study (GWAS) of mineral nutrient concentrations in the grains of 369 European wheat genotypes grown over three seasons. Data were analysed using two marker arrays: a 90K iSELECT Infinium array and a 35K Affymetrix SNP array based on best linear unbiased estimates (BLUEs). (A) Summary of the GWAS data for each mineral. The horizontal lines indicate the Bonferroni threshold (5.42). RAC875_c8642_231 is indicated. (B) Quantile-quantile plots showing the observed associations between SNPs and grain nutrient concentrations compared to expected associations.

**Fig. 3.**
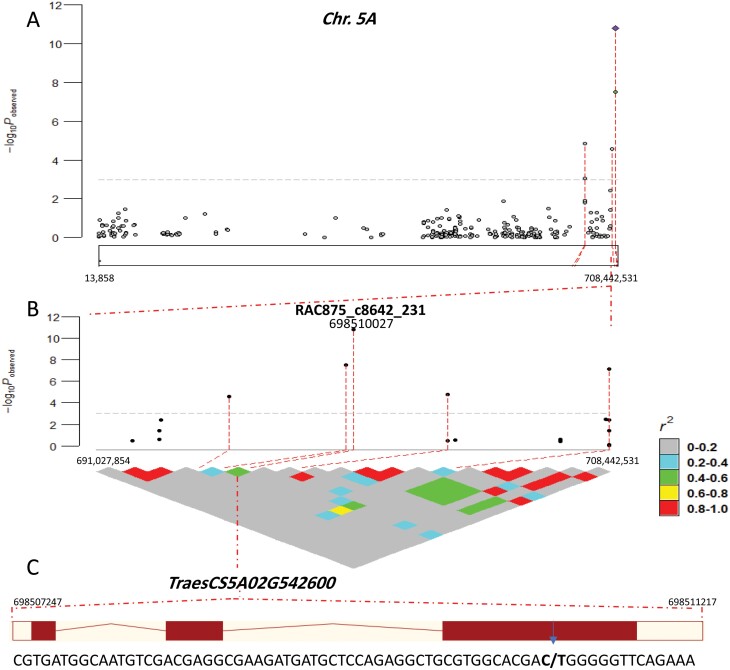

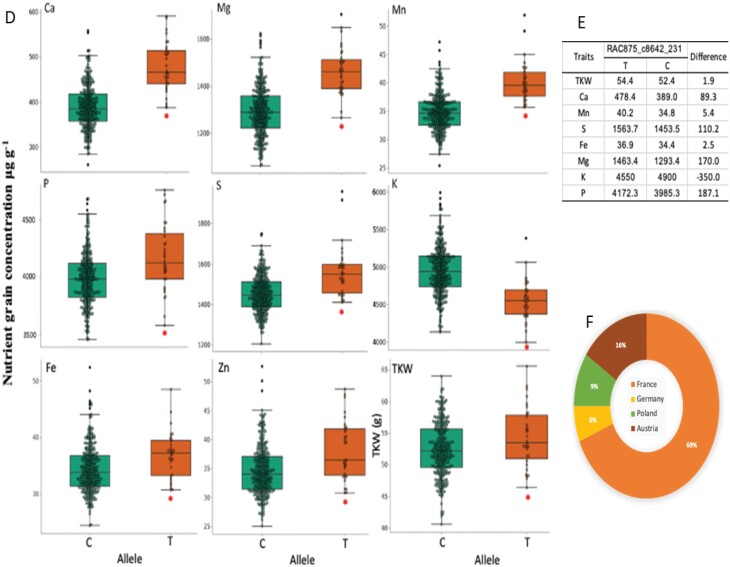
Marker–trait associations (MTAs) in chromosome 5A for nutrient mineral concentrations in grains of 369 European wheat genotypes grown over three seasons. (A) Manhattan plot for associations and (B) detail of the locus of the highly associated SNP RAC875_c8642_231. The heatmap below shows linkage disequilibrium for SNPs within 15 Mbp. (C) Structure of the candidate gene *TraesCS5A02G542600* showing the C/T sequence variation in third exon. (D) Box plots showing the allele effects on nutrient concentrations and thousand-kernel weight (TKW) in the genotypes, and (E) table showing the corresponding data for some of the traits. (F) Frequency distribution of countries of origin of the genotypes carrying the T allele.

GWAS analysis was performed to determine the genetic basis for the accumulation of the minerals in the grains using FarmCPU for 15 523 SNP markers. Most of the markers were mapped on the B-genome, followed by the A- and D-genomes. In brief, the marker–trait associations (MTAs) were distributed across the whole genome, except for 3D and 6D where 86 significant associations were detected [–log_10_(*P*)≥3], and the highest numbers of significant markers were found on 2B (12) followed by 5B (nine) and 3B (eight). The MTAs were identified using estimated BLUE values across the three years (Supplementary Table S4A). A total of 17 MTAs were above the Bonferroni threshold of –log_10_(*P*-value)=5.42 (Fig. 2A, Supplementary Table S4B). A total of 50 MTAs had positive effects and 36 had negative effects on the mineral concentrations (Supplementary Table S4A). The phenotypic variance explained by each SNP ranged from 0.04–10.54% (Supplementary Table S4A, B). RAC875_c8642_231 showed the highest –log_10_(*P*-value) of Ca, Mg, Mn, and K (Figs 2A, 3A) and had a positive effect on Ca, Mn, Mg, and S (26, 1.8, 49, and 28 μg g^–1^, respectively) and a negative effect on K (–117 μg g^–1^) (Supplementary Table S4B). This marker could explain ~10% of the variation in Ca and ~ 3.4% in K. This indicated that there was allelic variation at this marker among the genotypes that lead to variation in the mineral concentrations (Supplementary Table S5). The Q-Q plots showing the observed associations between SNPs and grain nutrient concentrations compared to expected associations are presented in Fig. 2B.

**Fig. 4.**
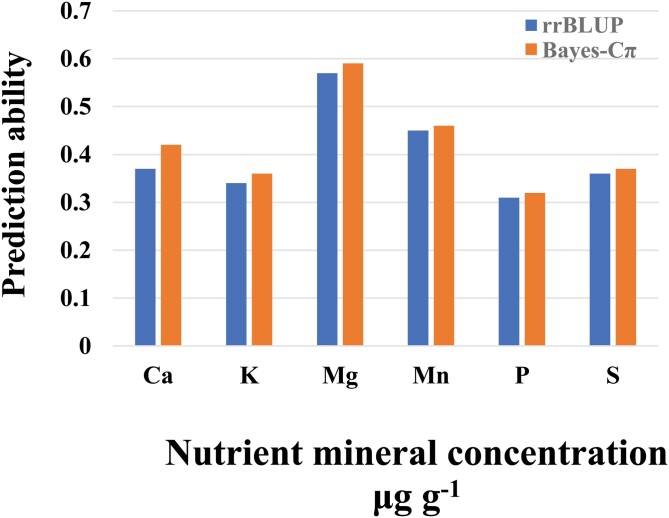
Genomic prediction ability for nutrient mineral concentrations in grains of 369 European wheat genotypes grown over three seasons according to best linear unbiased estimates (BLUEs) values as determined using two different statistical models, ridge regression–best linear unbiased prediction (RR-BLUP) and the Bayes-Cπ method.

Statistical methods for developing prediction models for the breeding value of studied traits include ridge regression–best linear unbiased prediction (RR-BLUP) and the Bayes-Cπ method. We found that the mean of prediction ability values that resulted from using these two different methods were in close agreement for the individual minerals. The highest values were obtained for Mg followed by Mn (Fig. 4).

### Detection of candidate genes and expression analysis

The significant SNPs that we identified were used to predict gene models located on the wheat genome using the reference Chinese Spring RefSeqv1.1. Candidate gene transcripts and their corresponding annotation information were obtained from EnsemblPlants (http://plants.ensembl.org/Triticum_aestivum). The results showed that the genes were annotated as transmembrane transporter activity, protein kinase, ATPase-coupled cation transmembrane transporter, metal ion binding, and magnesium ion binding. All the potential candidate genes and their corresponding annotations within all detected loci are listed in Table 1. It was notable that *TraesCS5A02G542600*, which was annotated as transmembrane transporter activity and that underlay the natural variation of Ca, Mn, Mg, S, and K, was located on chromosome 5A (698 507 247–698 511 217 bp; Fig. 3A). Interestingly, RAC875_c8642_231 that had the highest association value among all the SNPs was also located on chromosome 5A (698 510 016 bp), inside *TraesCS5A02G542600* (Fig. 3A). Based on the gene structure, the RAC875_c8642_231 marker was physically located inside exon 3 of *TraesCS5A02G542600* (Fig. 3C). Polymorphism analysis of RAC875_c8642_231 alleles showed that the genotypes of the population either carried the C allele (337 genotypes) or the T allele (32 genotypes) (Supplementary Table S5). The genotypes with the T allele had significantly higher (*P*<0.05) mineral concentration values in the grains except for K (Fig. 3D). A positive effect of the T allele on TKW was also found. The genotypes that carried the T allele originated from France (Fig. 3F) and showed the highest minerals concentration (Fig. 3E) .

**Table 1.** Candidate genes for the significant marker–trait associations for nutrient mineral concentrations in a panel of 369 European wheat genotypes

Chromosome	Mineral	Gene ID	Description	Putative functionality
5A	Ca, Mn, Mg, S, K	*TraesCS5A02G542600*	Transmembrane transporter activity	Major facilitator superfamily
3B	Ca	*TraesCS3B02G006700*	Transferase activity	Diacylglycerol O-acyltransferase
7A	Ca	*TraesCS7A02G169100*	Transmembrane transporter activity	WAT1-related protein
5B	Ca	*TraesCS5B02G403400*	Aspartate-semialdehyde dehydrogenase activity	Semialdehyde dehydrogenase
6B	Ca	*TraesCS6B02G428500*	NAD^+^ kinase activity	NAD kinase/diacylglycerol kinase-like domain superfamily
4B	K	*TraesCS4B02G380200*	Cysteine-type endopeptidase activity	Type II CAAX prenyl endopeptidase Rce1-like
5B	K	*TraesCS5B02G301100*	Protein binding	WD40 repeat
7D	K	*TraesCS7B02G478200*	DNA binding	Homeobox superfamily
	K	*TraesCS3B02G590500*	Protein kinase activity	Protein kinase superfamily
4A	K	*TraesCS4A02G352200*	Ubiquitin protein ligase activity	E3 ubiquitin-protein ligase RNF170
2D	K	*TraesCS2D02G190600*	Protein kinase activity	Protein kinase domain-containing protein
7D	K	*TraesCS7D02G540700*	DNA binding	Homeobox superfamily
3B	Mg	*TraesCS3B02G125400*	Membrane	Uncharacterized protein
3B	Mg	*TraesCS3A02G514300*	Protein binding	F-box superfamily
4A	Mg	*TraesCS4A02G369500*	Catalytic activity	Alkaline-phosphatase
4B	Mg	*TraesCS4B02G293600*	ATPase-coupled cation transmembrane transporter activity	P-type ATPase
5B	Mg	*TraesCS5B02G427800*	Protein binding	IQ motif, EF-hand binding site
7A	Mg	*TraesCS7A02G498400*	Metal ion binding	Ubiquitin specific protease
4B	Mn	*TraesCS4B02G024300*	Protein binding	Tetratricopeptide-like helical domain superfamily
5B	Mn	*TraesCS5B02G012300*	Magnesium ion binding	Phosphopyruvate hydratase
5B	Mn	*TraesCS5B02G042900*	Protein binding	Tetratricopeptide-like helical domain superfamily
6B	Mn	*TraesCS6B02G181100*	Integral component of membrane	Uncharacterized protein
2A	P	*TraesCS2A02G130200*	Hydrolase activity	Haloacid Dehalogenase
2A	P	*TraesCS2A02G123400*	Oxidoreductase activity	FAD-binding PCMH-type domain-containing protein
2B	P	*TraesCS2B02G202600*	Integral component of membrane	GPI transamidase subunit PIG-U
3B	P	*TRAES_3BF053100070CFD_c1* *TraesCS3B02G013300*	Cytoskeleton	Targeting protein for Xklp2 domain containing protein, expressed
3B	P	*TraesCS3B02G125400*	Integral component of membrane	Uncharacterized protein
4A	P	*TraesCS4A02G369500*	Catalytic activity	Alkaline-phosphatase
5A	P	*TraesCS5A02G486100*	Calmodulin binding	CALMODULIN-BINDING PROTEIN60
6A	P	*TraesCS6A02G352300*	Protein binding	Ankyrin repeat
6B	P	*TraesCS6B02G002500*	Protein kinase activity	Serine-threonine/tyrosine-protein kinase
1A	S	*TraesCS1A02G292100*	Protein kinase activity	Serine-threonine/tyrosine-protein kinase
1A	S	*TraesCS1A02G322300*	Serine-type endopeptidase activity	Peptidase S9
1B	S	*TraesCS1B02G338500*	Cysteine-type peptidase activity	Papain-like cysteine peptidase superfamily
1B	S	*TraesCS1B02G033200*	Cysteine-type peptidase activity	Papain-like cysteine peptidase superfamily
2B	S	*TraesCS2B02G166300*	Regulation of DNA methylation	SAC3 family protein B
2B	S	*TraesCS2B02G169300*	Lipid binding	Synaptotagmin-like mitochondrial-lipid-binding domain
2B	S	*TraesCS2B02G169500*	Nucleic acid binding	Cold-shock protein, DNA-binding
2B	S	*TraesCS2B02G169400*	Actin binding	Stomatal closure-related actin-binding protein
2B	S	*TraesCS2B02G169400*	Actin binding	Stomatal closure-related actin-binding protein
2B	S	*TraesCS2B02G169500*	Nucleic acid binding	Cold-shock protein, DNA-binding
2D	S	*TraesCS2D02G074700*	Hydrolase activity	Amidohydrolase
4B	S	*TraesCS4B02G278100*	DNA-binding transcription factor activity	Heat shock transcription factor HsfA2-8
5B	S	*TraesCS5B02G392500*	DNA binding	B3 DNA binding domain
6A	S	*TraesCS6A02G037800*	Metal ion binding	Ribonuclease Nob1
7A	S	*TraesCS7A02G419800*	ADP binding	P-loop containing nucleoside triphosphate hydrolase

The significant SNPs identified in the current study were used to predict gene models located on the wheat genome using the reference Chinese Spring RefSeqv1.1. Candidate gene transcripts and their corresponding annotation information were obtained from the website of EnsemblPlants (http://plants.ensembl.org/Triticum_aestivum).

**Fig. 5.**
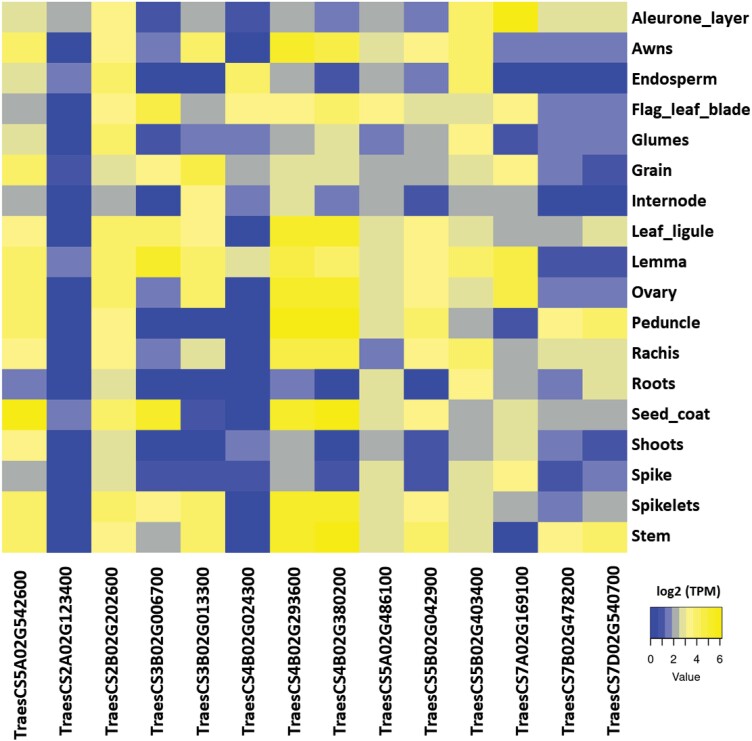
Expression patterns of selected candidate genes for mineral nutrient concentrations within different tissues of wheat. Expression data were obtained from the Wheat Gmap database (https://www.wheatgmap.org/expression/search/gene/) and are presented as a heatmap of transcripts per kilobase million (TPM) values.

We found a wide range of expression for the candidate genes in different grain tissues and at different developmental stages using the WheatGmap web tool (Fig. 5). Generally, *TraesCS2B02G202600* at 2B and *TraesCS5A02G542600* at 5A showed the highest expression in most of the organs and tissues, indicating that they play vital roles during growth, filling, and development. *TraesCS3B02G006700* was highly expressed in the lemma and seed coat, and also in the flag-leaf blade, leaf ligule, and grain. One of the significantly associated genes, *TraesCS6B02G002500*, showed very low expression in the tissues compared to the other genes and was removed from the expression analysis.

## Discussion

### Phenotypic variations in grain mineral across genotypes

The use of genomics breeding tools to increase the contents of essential nutrients in wheat grain is a promising approach for crop improvement, and the detection of natural phenotypic variation in existing genotypes is the primary step in this process (Yu and Tian, 2018). Overall, we observed wide natural variation in grain concentrations of Ca, Mg, Mn, P, K, and S in our panel of 369 European elite registered varieties (Fig. 1A, Supplementary Fig. S1), which was consistent with previous results (Bhatta *et al.*, 2018). We found high broad-sense heritability values for the mineral concentrations (*H*^2^>0.7; Supplementary Table S3), indicating that the major part of the variation was genetically controlled. Similar findings Ca, Mg and S for have been reported in recombinant inbred lines of tetraploid wheat (Peleg *et al.*, 2009), while lower *H*^2^ values have been found for Mn, P, and K in a wild barley NAM population (Herzig *et al.*, 2019). The positive correlations that we found among the minerals (Fig. 1B) suggested the presence of common genetic factors affecting the accumulation of these nutrients in the grains, and similar trends of correlations have been observed previously in hexaploid wheat (Shi *et al.*, 2013). The lack of correlations between thousand-kernel weight and the mineral contents implied that there was no clear effect of grain weight on the content.

### GWAS analysis and genomic prediction

Most of the previous genetic studies in wheat have used QTL mapping to investigate the genetic basis of nutrient accumulation (Shi *et al.*, 2013; Pu *et al.*, 2014). In these studies, bi-parental crosses have been used to identify QTLs and genes associated with the mineral concentrations. However, the resulting mapping is relatively low resolution and hence GWAS is increasingly being used as an alternative that can identify alleles within a relatively broader set of germplasm with high resolution (Alomari *et al.*, 2017, 2018, 2019; Alqudah *et al.*, 2020). In addition, GWAS analysis is useful for identifying molecular markers that are tightly linked to the genomic regions that underlie natural variation in nutrients, which can then be used in genomics-assisted breeding for enhancing the efficiency of biofortification (Collard *et al.*, 2005).

Most of the association mapping studies that have been undertaken for different complex traits have used single-locus GWAS models (GLM, MLM) and these require additional corrections, for example multiple testing corrections to control false positives (Yu *et al.*, 2006; Price *et al.*, 2010; Pujar *et al.*, 2020). However, the corrections are often either not stringent enough, or too stringent and overcompensate for population structure and kinship, which can lead to over-correction that results in some potentially important marker–trait associations (MTAs) being missed (Alqudah *et al.*, 2020). To help overcome this issue, a GAPIT model known as ‘fixed and random model circulating probability unification’ (FarmCPU) has been developed and has been shown to be much more powerful, efficient, and superior to previous approaches in reducing false positive/negative associations, particularly in complex traits, by incorporating multiple markers simultaneously as covariates in a stepwise MLM to partially remove confounding effects between testing markers and kinship (Liu *et al.*, 2016). For this reason, we applied the FarmCPU model in our GWAS analysis.

We observed many genomic regions harboring markers that were associated with nutrient concentrations. In total, we detected 19 MTAs for S, 15 for K, 15 for P, 14 for Mg, 13 for Ca, and 10 for Mn (Supplementary Table S4A). The most significant association was detected at the end of the long arm of chromosome 5A and was linked with the RAC875_c8642_231 marker at the position ∼114.5 cM (698 5100 16 bp; Fig. 3). This was linked with all the minerals that we focused on except for P. P is positively correlated with phytate content in plants and it is known as an ‘anti-nutrient’ compound that negatively influences the absorption of other minerals in the human body (Stangoulis *et al.*, 2007). The RAC875_c8642_231 marker therefore appears to be a good candidate in breeding wheat to improve the concentrations of many minerals within the grain simultaneously. We also observed that the K concentration in the grain was negatively correlated with the concentrations of Ca, Mn, Mg, and S (Fig. 1B). MTAs for grain K concentration were also negatively associated with those for Ca, Mn, Mg, and S at the 5A locus (Supplementary Table S4A.

Co-localization of QTLs on chromosome 5A of wheat have previously been reported for N, Fe, Cu, Mg, and K (Shi *et al.*, 2013), and Fe, Zn, Cu, and Mg but not P (Cu *et al.*, 2020), and the latter study detected a co-located locus for Zn, Fe, Cu, P, and Mn on chromosome 5B at ~95 cM. It therefore seems that the QTL on chromosome 5A plays a vital role in mineral accumulation in wheat, and this needs further functional validation. Our results showed that most of the genotypes with the highest nutrient contents originated from France (Fig. 1E), and these were characterized by having awns. The top five of these genotypes were Isengrain, Inoui, Nirvana, Exotic, and Lona (Supplementary Table S1), and including varieties such as this in breeding programs might help in enhancing the accumulation of minerals in wheat grain. We extended our analysis to include the genomic prediction (GP) method, which has a practical role in improving the breeding efficiency of quantitative and complex traits. The predictability results for our wheat panel showed low-to-moderate values (Fig. 4), which was in agreement with a previous study of macro- and micro-nutrients in wheat (Manickavelu *et al.*, 2017). GP might be considered as a promising approach for enhancing nutrient minerals in wheat in situations where relatively large germplasm panels are used with high numbers of markers, thus giving more accurate estimates of breeding values.

### Candidate genes and their expression patterns

The recently released IWGSC RefSeq v1.1 genome by EnsemblPlants enabled us to further investigate the candidate genes potentially responsible for the variations in the grain nutrient concentrations (listed in Table 1). In the following sections we only consider high-confidence candidate genes that are linked to transcription factor regulators, transporters, and grain development.

#### Ca.

The most significant MTA detected in the GWAS output was found to be associated with Ca, K, Mg, Mn, and S, and was located on chromosome 5A (114.5 cM; Fig. 3). It was linked to the gene *TraesCS5A02G542600*, which encodes a transmembrane transporter belonging to the Major Facilitator Superfamily (MSF). This gene family is known to be one of the two largest superfamilies of membrane transporters, and they act as uniporters or symporters for different substances (Niño-González *et al.*, 2019). Interestingly, three recent studies in wheat have explored the role of *TraesCS5A02G542600* in the inhibition of awn formation; however, whilst it is located at the genetic locus of awn suppression, a closely linked gene has been considered as the most likely candidate (DeWitt *et al.*, 2020; Huang *et al.*, 2020; Würschum *et al.*, 2020). Further studies are needed to shed light on the role of *TraesCS5A02G542600* in nutrient mineral uptake and accumulation in wheat grains. We found that the expression of *TraesCS5A02G542600* was very high in grain tissues such as the seed coat, lemma, aleurone, and endosperm, and in tissues closely associated with the grain such as the peduncle, spikes, and spikelets (Fig. 5), which would be consistent with its involvement in mineral accumulation in the grains.

Another candidate gene for Ca accumulation was *TraesCS7A02G169100*, (125 712 948–125 715 936 bp), which has an annotation as a transmembrane transporter and encodes a WALLS ARE THIN 1 (WAT1)-related protein. This gene has been found to be involved in secondary cell wall formation (Kaur *et al.*, 2017), and it has been demonstrated that Ca^2+^ plays a major role in determining the structural rigidity of the cell wall, with high concentrations making the wall more rigid and less plastic whilst low concentrations make the wall more pliable and easily ruptured (Hepler, 2005). *TraesCS7A02G169100* was highly expressed in the aleurone layer, the lemma, and within the grain (Fig. 5).

*TraesCS3B02G006700* (3 601 450–3 607208 bp) was annotated as transferase activity and it encodes a diacylglycerol O-acyltransferase that catalyses the final step of the triacylglycerol (TAG) synthesis. TAG shows a significant increase in accumulation in Arabidopsis seedlings during nitrogen deprivation (Yang *et al.*, 2011), and it is therefore possible that it might have a role in the accumulation of other nutrient minerals such as Ca in wheat grains. Expression of *TraesCS3B02G006700* was found to be high for the lemma, seed coat, flag-leaf blade, leaf ligule, and grain (Fig. 5), which potentially supports this hypothesis.

*TraesCS5B02G403400* (580 100 440–580 104 246bp) encodes semialdehyde dehydrogenase, which is one of three enzymes constituting the gamma-aminobutyric acid shunt, a metabolic pathway that has been associated with abiotic stress responses in durum wheat (Carillo, 2018). The first enzyme in the pathway is glutamate decarboxylase (GAD), which is a calcium/calmodulin-binding protein (Baum *et al.*, 1996; Busch and Fromm, 1999).

#### K.

Two Homeobox superfamily genes, *TraesCS7B02G478200* (733 527 123–733 530 917 bp) and *TraesCS7D02G540700* (630 527 242–630 530 005 bp) were linked with K, and this superfamily is one of the transcription factor families that are involved in plant development, growth, and in the response to diverse stresses (Wei *et al.*, 2019). These genes showed almost the same expression pattern with the highest value in the peduncle followed by the stem, rachis, aleurone layer, seed coat, leaf ligule, and spikelets (Fig. 5).

*TraesCS3B02G590500* (815 476 561–815 480 015 bp) and *TraesCS2D02G190600* (134 638 904–134 645 251 bp) were both annotated as protein kinase, which is a large superfamily that plays vital roles in plant development and stress tolerance; however, the functions of only a limited number of protein kinases have been studied in wheat (Wei and Li, 2019 Several studies have shown that the E3 ubiquitin-protein ligase gene *TraesCS4A02G352200* (627 813 928–627 816 836 bp) is associated with the uptake and accumulation of various minerals such as Mn, Zn, and P in wheat and pearl millet (Cu *et al.*, 2020; Pujar *et al.*, 2020), and thus it might have a role in K uptake as well.

#### Mg.

We detected *TraesCS4B02G293600* (579 391 172–579 398 644 bp) encoding a P-type ATPase on chromosome 4B, and interestingly this is considered as a transmembrane protein that plays a crucial role in the transport of a wide variety of cations across membranes and is vital for ion homeostasis and detoxification of heavy metals (Axelsen and Palmgren, 2001). Very high expression levels were detected for in the peduncle, seed coat, spikelets, leaf ligule, and stem (Fig. 5).

*TraesCS7A02G498400* (688 960 269–688 968 295 bp) encoding ubiquitin specific protease was detected on chromosome 7A and annotated as metal ion binding.

#### Mn.

Genes encoding tetratricopeptide-like helical domain superfamily proteins, namely *TraesCS4B02G024300* (173 621 92–173 688 28 bp and *TraesCS5B02G042900* (475 842 53–475 880 87 bp), were found on chromosomes 4B and 5B, respectively. These are involved in root development and auxin polar transport as well as gibberellin signal transduction in Arabidopsis (Jacobsen *et al.*, 1996; Zhang *et al.*, 2015), which might indicate their involvement in Mn accumulation in wheat plants. These two genes had their highest expression in the endosperm, flag-leaf blade, lemma, grain, and glumes.

 Another gene was identified on chromosome 5B as encoding phosphopyruvate hydratase and annotated as magnesium ion binding, namely *TraesCS5B02G012300* (12 324 287–12 328 841 bp). (Silva *et al.*, 2018) have reported an association between phosphopyruvate hydratase and enhanced nitrogen metabolism in maize seedlings.

#### P.

*TraesCS2A02G130200* (77 938 466–77 941 846 bp) encoding a haloacid dehalogenase (HAD) was found on chromosome 2A. HAD enhances phosphate accumulation, and *LePS2* in tomato was the first low-Pi inducible gene in this superfamily to be characterized (Baldwin *et al.* 2001, 2008). Another HAD gene, *PvHAD1*, shows specific induction at low P and encodes a functional serine/threonine phosphatase (Liu *et al.*, 2012). Two other HAD genes responsive to low P, *AtPPsPase1* and *AtPECP1*, have been reported to encode functional pyrophosphatase and phosphoethanolamine phosphatase in Arabidopsis (Pandey *et al.*, 2017). In rice, only two HAD genes have been shown to be up-regulated under P deficiency, namely *OsACP1* (Hur *et al.*, 2007) and *OsHAD1* (Pandey *et al.*, 2017) . These studies therefore suggest HAD superfamily members have important functions in P accumulation in various plants; however, there are no studies available on wheat.

*TraesCS2A02G123400* (729 311 06–729 331 77 bp) encoding a FAD-binding PCMH-type domain-containing protein was detected on chromosomes 2A, and *TraesCS2B02G202600* (182 355 759–182 361 233 bp) encoding GPI transamidase subunit PIG-U was detected on chromosome 2B, which may be involved in P accumulation in wheat.

Interestingly, *TraesCS3B02G01330* (5 952 761–5 955 519 bp) encoding a targeting protein for Xklp2 (TPX2) was detected on chromosome 3B. This gene is important for phosphorylation/dephosphorylation of regulatory proteins by phosphatase. The *TPX2* gene is not well studied, and it is only recently that two genes, *TPXL2* and *TPXL3*, have been functionally characterized in Arabidopsis (Dvořák Tomaštíková *et al.*, 2020). Another gene involved in phosphorylation, *TraesCS6B02G002500* (1 963 516–1 970 774 bp), was detected on chromosome 6B and encodes as serine-threonine/tyrosine-protein kinase (Dvořák Tomaštíková *et al.*, 2020). Another important gene, *TraesCS4A02G369500* (641 504 088–641 508 259 bp), was detected on chromosome 4A and belongs to the alkaline-phosphatase family, which is and associated with phosphate efflux from arbuscules (Aono *et al.*, 2004). In addition, *TraesCS5A02G486100* (657 191 801–657 195 889 bp) encoding CALMODULIN-BINDING PROTEIN60 has a significant role in protein phosphorylation/dephosphorylation (Bergey *et al.*, 2014).

#### S.

Two SNP markers were detected on chromosome 1B as papain-like cysteine peptidase (PLCP) superfamily genes, namely *TraesCS1B02G338500* (566 601 144–566 605 225 bp) and *TraesCS1B02G033200* (162 164 83–162 176 35 bp). PLCPs have important functions in plant growth, seed germination, anther development, and senescence. Thus, they might play a role in S accumulation in wheat grains (Liu *et al.*, 2018).

### Conclusions

There have been very few GWAS analyses identifying significant loci associated with nutrient mineral accumulation in wheat grains. Hence, the marker–trait associations and candidate genes identified in this study will be useful for the future genetic improvement of wheat nutritional quality through marker-assisted selection. This study also provides useful information on the range of phenotypic variation encountered within European wheat germplasm. However, further research is needed for a better understanding of the relationships between the individual mineral nutrients in wheat and to provide more detailed information that can lead to efficient breeding to overcome malnutrition problems.

## Supplementary data

The following supplementary data are available at *JXB* online.

Fig. S1. Frequency distributions of nutrient mineral concentrations in wheat grains based on BLUEs.

Table S1. Details of the 369 European wheat varieties used in the study.

Table S2. Phenotypic variation for grain nutrient concentrations across the three study years and BLUE values.

Table S3. ANOVA results and heritability values for the nutrient concentrations.

Table S4. Significant marker–trait associations of the SNP markers based on BLUE data, and those above the Bonferroni correction threshold.

Table S5. Allelic variation analysis for the RAC875_c8642_231 marker across all the genotypes.

Table S6. Expression analysis for the putative candidate genes underlying the accumulation of nutrient minerals in the grains.

erab297_suppl_Supplementary_Figure_S1_Table_S1-S6

## Data Availability

All data supporting the findings of this study are available within the paper and within its supplementary materials published online.
