# Supplementary material for: Toward identification of a putative candidate gene for nutrient mineral accumulation in wheat grains for human nutrition purposes
Source: J Exp Bot. 2021 Jun 18;72(18):6305–18. doi: 10.1093/jxb/erab297 (PMC8483787; doi:10.1093/jxb/erab297)
Supplement: erab297_suppl_Supplementary_Figure_S1_Table_S1-S6 [file erab297_suppl_supplementary_figure_s1_table_s1-s6.pdf]

A Major Facilitator Superfamily Transporter is a putative candidate gene for nutrient mineral accumulation in bread wheat grains

Authors: Dalia Z. Alomari[1*], Ahmad M. Alqudah[2], Klaus Pillen[2], Nicolaus von Wirén[1], Marion S. Röder[1]

[1]Leibniz Institute of Plant Genetics and Crop Plant Research (IPK), Corrensstrasse 3, D-06466 Stadt Seeland OT Gatersleben, Germany
[2]Institute of Agricultural and Nutritional Sciences, Martin-Luther-University Halle-Wittenberg, Betty-Heimann-Str. 3, 06120 Halle/Saale, Germany

*Corresponding author:
Dalia Z. Alomari: alomari@ipk-gatersleben.de / alamridalia@gmail.com

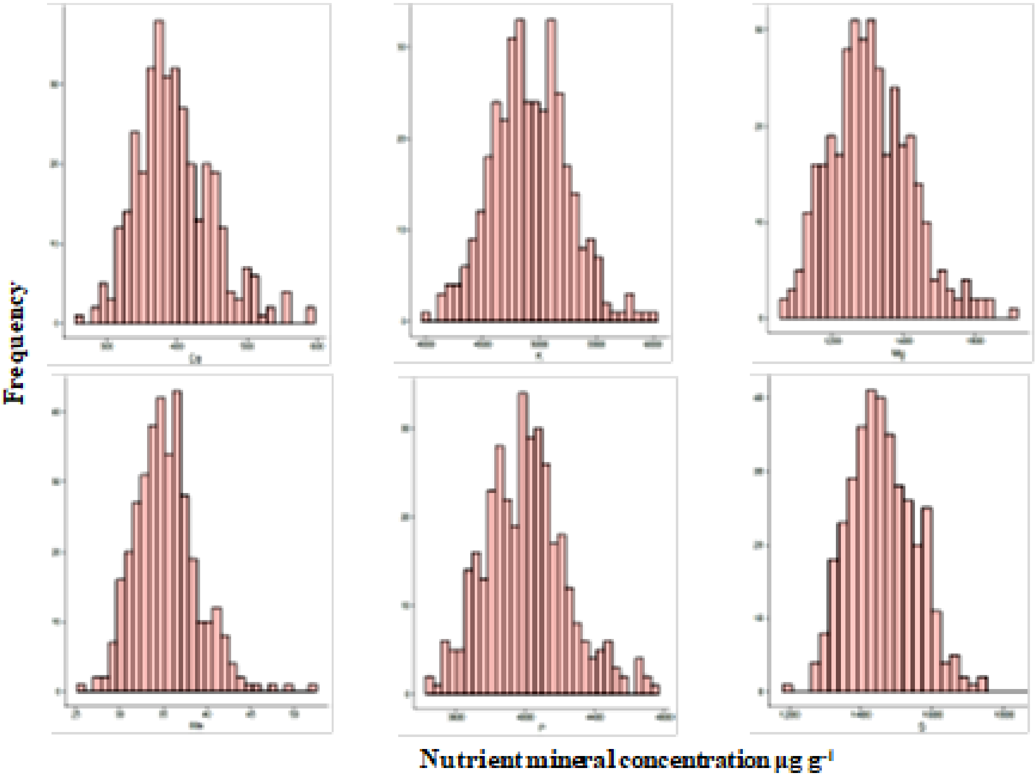

Figure (S1): The phenotypic distribution of nutrient minerals concentration in wheat grains based on BLUEs.

Supplementary table S1: The detailed information of 369 European wheat varieties.

Genotype number	Name	Type	Origin**
GW0001	Actros	Winter wheat	**D**
GW0002	Akratos	Winter wheat	**D**
GW0003	Akteur	Winter wheat	**D**
GW0004	Alidos	Winter wheat	**D**
GW0005	Altos	Winter wheat	**D**
GW0006	Anthus	Winter wheat	**D**
GW0007	Asketis	Winter wheat	**D**
GW0008	Astron	Winter wheat	**D**
GW0009	Atlantis	Winter wheat	**D**
GW0010	Batis	Winter wheat	**D**
GW0011	Biscay	Winter wheat	**D**
GW0012	Boomer	Winter wheat	**D**
GW0013	Borneo	Winter wheat	**D**
GW0014	Brilliant	Winter wheat	**D**
GW0015	Bussard	Winter wheat	**D**
GW0016	Buteo	Winter wheat	**D**
GW0017	Campari	Winter wheat	**F**
GW0018	Capnor	Winter wheat	**A**
GW0019	Cardos	Winter wheat	**D**
GW0021	Centrum	Winter wheat	**D**
GW0022	Certo	Winter wheat	**D**
GW0023	Cetus	Winter wheat	**D**
GW0024	Compliment	Winter wheat	**D**
GW0025	Contra	Winter wheat	**D**
GW0026	Cubus	Winter wheat	**D**
GW0027	Dekan	Winter wheat	**D**
GW0028	Discus	Winter wheat	**D**
GW0029	Drifter	Winter wheat	**D**
GW0030	Elegant	Winter wheat	**D**
GW0031	Ellvis	Winter wheat	**D**
GW0032	Enorm	Winter wheat	**D**
GW0033	Esket	Winter wheat	**D**
GW0034	Fedor	Winter wheat	**D**
GW0035	Flair	Winter wheat	**D**

GW0036	Format	Winter wheat	**D**
GW0037	Greif	Winter wheat	**D**
GW0038	Haldor	Winter wheat	**D**
GW0039	Hermann	Winter wheat	**D**
GW0040	Heroldo	Winter wheat	**D**
GW0041	Impression	Winter wheat	**D**
GW0042	Inspiration	Winter wheat	**D**
GW0043	Jenga	Winter wheat	**D**
GW0044	Koch	Winter wheat	**D**
GW0045	Kontrast	Winter wheat	**D**
GW0046	Kranich	Winter wheat	**D**
GW0047	Leiffer	Winter wheat	**D**
GW0048	Limes	Winter wheat	**D**
GW0049	Lucius	Winter wheat	**D**
GW0050	Ludwig	Winter wheat	**D**
GW0051	Magister	Winter wheat	**D**
GW0052	Magnus	Winter wheat	**D**
GW0053	Manager	Winter wheat	**D**
GW0054	Mandub	Winter wheat	**D**
GW0055	Manhattan	Winter wheat	**D**
GW0056	Maverick	Winter wheat	**D**
GW0057	Meteor	Winter wheat	**D**
GW0058	Meunier	Winter wheat	**D**
GW0059	Milvus	Winter wheat	**D**
GW0060	Mirage	Winter wheat	**D**
GW0061	Monopol	Winter wheat	**D**
GW0062	Mulan	Winter wheat	**D**
GW0064	Naturastar	Winter wheat	**D**
GW0065	Olivin	Winter wheat	**D**
GW0066	Opus	Winter wheat	**D**
GW0067	Paroli	Winter wheat	**D**
GW0068	Petrus	Winter wheat	**D**
GW0069	Piko	Winter wheat	**D**
GW0070	Potenzial	Winter wheat	**D**
GW0071	Privileg	Winter wheat	**D**
GW0072	Quebon	Winter wheat	**D**
GW0073	Retro	Winter wheat	**D**
GW0074	Ritmo	Winter wheat	**D**
GW0075	Romanus	Winter wheat	**D**
GW0076	Schamane	Winter wheat	**D**

GW0077	Skagen	Winter wheat	**D**
GW0078	Skalmeje	Winter wheat	**D**
GW0079	Skater	Winter wheat	**D**
GW0080	Sobi	Winter wheat	**D**
GW0081	Sokrates	Winter wheat	**D**
GW0082	Solitär	Winter wheat	**D**
GW0083	Striker	Winter wheat	**D**
GW0084	SW Topper	Winter wheat	**D**
GW0085	Tambor	Winter wheat	**D**
GW0086	Tarso	Winter wheat	**D**
GW0087	Tommi	Winter wheat	**D**
GW0088	Toras	Winter wheat	**D**
GW0089	Toronto	Winter wheat	**D**
GW0090	Transit	Winter wheat	**D**
GW0091	Tuareg	Winter wheat	**D**
GW0092	Tukan	Winter wheat	**D**
GW0093	Tulsa	Winter wheat	**D**
GW0094	Türkis	Winter wheat	**D**
GW0095	Winnetou	Winter wheat	**D**
GW0096	Zentos	Winter wheat	**D**
GW0097	Zobel	Winter wheat	**D**
GW0098	Cliff	Winter wheat	**DK**
GW0099	Dream	Winter wheat	**D**
GW0100	Florett	Winter wheat	**DK**
GW0101	History	Winter wheat	**D**
GW0102	Lindos	Winter wheat	**_**
GW0103	Julius	Winter wheat	**D**
GW0104	Tiger	Winter wheat	**D**
GW0105	Exotic	Winter wheat	**F**
GW0106	Andalou	Winter wheat	**F**
GW0107	LOCH 3754 Adlon	Winter wheat	**D**
GW0109	Acienda	Winter wheat	**F**
GW0110	Aguila	Winter wheat	**F**
GW0111	Alcazar	Winter wheat	**F**
GW0113	Allister	Winter wheat	**F**
GW0114	Arack	Winter wheat	**F**
GW0115	Arobase	Winter wheat	**F**
GW0116	Astuce	Winter wheat	**F**
GW0117	Attlass	Winter wheat	**F**
GW0118	Aubusson	Winter wheat	**F**

GW0119	Autan	Winter wheat	**F**
GW0120	Avantage	Winter wheat	**F**
GW0121	Azimut	Winter wheat	**F**
GW0122	Azzuro	Winter wheat	**F**
GW0123	Bagatelle 007	Winter wheat	**F**
GW0124	Balance	Winter wheat	**F**
GW0125	Baltimor	Winter wheat	**F**
GW0126	Bastide	Winter wheat	**F**
GW0127	Boston	Winter wheat	**F**
GW0128	Brando	Winter wheat	**F**
GW0129	Calisto	Winter wheat	**D**
GW0130	Campero	Winter wheat	**F**
GW0131	Caphorn	Winter wheat	**D**
GW0132	Caribou	Winter wheat	**F**
GW0133	Catalan	Winter wheat	**F**
GW0134	Cezanne	Winter wheat	**F**
GW0135	Chagall	Winter wheat	**F**
GW0136	Charger	Winter wheat	**F**
GW0137	Craklin	Winter wheat	**F**
GW0138	Dinosor	Winter wheat	**F**
GW0140	Ephoros	Winter wheat	**F**
GW0141	Epidoc	Winter wheat	**F**
GW0142	Equilibre	Winter wheat	**F**
GW0143	Equinox	Winter wheat	**F**
GW0144	Eveil	Winter wheat	**F**
GW0145	Exotic	Winter wheat	**F**
GW0146	Folio	Winter wheat	**F**
GW0147	Forban	Winter wheat	**F**
GW0148	Garcia	Winter wheat	**F**
GW0149	Graindor	Winter wheat	**F**
GW0150	Grisby	Winter wheat	**F**
GW0151	Hamac	Winter wheat	**F**
GW0152	Haussmann	Winter wheat	**F**
GW0153	Hourra	Winter wheat	**F**
GW0155	Incisif	Winter wheat	**F**
GW0156	Inoui	Winter wheat	**F**
GW0157	Instinct	Winter wheat	**F**
GW0158	Intact	Winter wheat	**F**
GW0159	Intense	Winter wheat	**F**
GW0160	Isengrain	Winter wheat	**F**

GW0161	Kleber	Winter wheat	**F**
GW0162	Levis	Winter wheat	**F**
GW0163	Lona	Winter wheat	**F**
GW0164	Macro	Winter wheat	**UK**
GW0165	Marcheval	Winter wheat	**F**
GW0166	Maxyl	Winter wheat	**F**
GW0167	Mendel	Winter wheat	**F**
GW0168	Mercato	Winter wheat	**F**
GW0169	Nirvana	Winter wheat	**F**
GW0170	Nuage	Winter wheat	**F**
GW0171	Octet	Winter wheat	**F**
GW0172	Ordeal	Winter wheat	**F**
GW0173	Ornicar	Winter wheat	**F**
GW0174	Orpic	Winter wheat	**F**
GW0175	Orvantis	Winter wheat	**F**
GW0176	Paladain	Winter wheat	**F**
GW0177	Paledor	Winter wheat	**F**
GW0178	Parador	Winter wheat	**F**
GW0180	PR 22 R 28	Winter wheat	**F**
GW0181	PR22R20	Winter wheat	**F**
GW0182	Pulsar	Winter wheat	**F**
GW0183	Pytagor	Winter wheat	**F**
GW0184	Quatuor	Winter wheat	**F**
GW0185	Raison	Winter wheat	**F**
GW0186	Richepain	Winter wheat	**F**
GW0187	Rosario	Winter wheat	**F**
GW0188	Royssac	Winter wheat	**F**
GW0189	Runal	Winter wheat	**F**
GW0190	Samurai	Winter wheat	**F**
GW0191	Sankara	Winter wheat	**F**
GW0192	Scorpion 25	Winter wheat	**F**
GW0193	Semafor	Winter wheat	**F**
GW0194	Seyrac	Winter wheat	**F**
GW0195	Sisley	Winter wheat	**F**
GW0196	Sisley	Winter wheat	**F**
GW0197	Tamaro	Winter wheat	**F**
GW0198	Tapidor	Winter wheat	**F**
GW0199	Titlis	Winter wheat	**F**
GW0200	Toisondor	Winter wheat	**F**
GW0201	Trocadero	Winter wheat	**F**

GW0202	Versailles	Winter wheat	**F**
GW0203	CCB Ingénio	Winter wheat	**E**
GW0204	Inédit	Winter wheat	**F**
GW0205	CCB Préférence	Winter wheat	**F**
GW0206	Intérêt	Winter wheat	**F**
GW0207	Expert	Winter wheat	**F**
GW0209	H05581A	Winter wheat	**F**
GW0210	H04438	Winter wheat	**F**
GW0211	H03311	Winter wheat	**F**
GW0212	H03309	Winter wheat	**F**
GW0213	Innov	Winter wheat	**F**
GW0214	Oratorio	Winter wheat	**F**
GW0215	Recital	Winter wheat	**F**
GW0216	PR22R58	Winter wheat	**F**
GW0217	Premio	Winter wheat	**F**
GW0218	Bermude	Winter wheat	**F**
GW0219	Euclide	Winter wheat	**F**
GW0220	Aldric	Winter wheat	**F**
GW0221	Altigo	Winter wheat	**F**
GW0222	Bagou	Winter wheat	**F**
GW0223	Boisseau	Winter wheat	**F**
GW0224	Iridium	Winter wheat	**F**
GW0225	Andi	Winter wheat	_
GW0226	Ambition	Winter wheat	**DK**
GW0228	Bill	Winter wheat	**DK**
GW0229	Cassiopeia	Winter wheat	**DK**
GW0230	Contur	Winter wheat	**DK**
GW0231	Fastnet	Winter wheat	**DK**
GW0232	Frument	Winter wheat	**DK**
GW0233	Hereford	Winter wheat	**DK**
GW0234	Hereward	Winter wheat	**UK**
GW0235	Portland	Winter wheat	**DK**
GW0236	Senat	Winter wheat	**DK**
GW0237	Smuggler	Winter wheat	**DK**
GW0238	Solist	Winter wheat	**DK**
GW0239	Symbol	Winter wheat	**DK**
GW0240	Torkil	Winter wheat	**DK**
GW0241	Trintella	Winter wheat	**DK**
GW0242	Tuscan	Winter wheat	**DK**
GW0243	Zanatan	Winter wheat	**DK**

GW0244	Achat	Winter wheat	**A**
GW0245	Antonius	Winter wheat	**A**
GW0246	Astrado	Winter wheat	**A**
GW0247	Capo	Winter wheat	**D**
GW0248	Element	Winter wheat	**A**
GW0249	Eriwan	Winter wheat	**A**
GW0250	Eurojet	Winter wheat	**A**
GW0251	Fridolin	Winter wheat	**A**
GW0252	Globus	Winter wheat	**A**
GW0253	Rainer	Winter wheat	**A**
GW0254	Vitus	Winter wheat	**_**
GW0255	Xenos	Winter wheat	**A**
GW0256	Apache	Winter wheat	**F**
GW0258	Banquet	Winter wheat	**CZ**
GW0259	Corsaire	Winter wheat	**CZ**
GW0260	Estica	Winter wheat	**CZ**
GW0262	Semper	Winter wheat	**CZ**
GW0263	Tower	Winter wheat	**CZ**
GW0264	Batuta	Winter wheat	**PL**
GW0265	Bogatka	Winter wheat	**PL**
GW0266	Figura	Winter wheat	**PL**
GW0267	Finezja	Winter wheat	**PL**
GW0268	Fregata	Winter wheat	**PL**
GW0269	Izyda	Winter wheat	**PL**
GW0270	Kobiera	Winter wheat	**PL**
GW0271	Kobra Plus	Winter wheat	**PL**
GW0272	Korweta	Winter wheat	**PL**
GW0273	Legenda	Winter wheat	**PL**
GW0274	Markiza	Winter wheat	**PL**
GW0275	Mewa	Winter wheat	**PL**
GW0276	Muza	Winter wheat	**PL**
GW0277	Nadobna	Winter wheat	**PL**
GW0278	Naridana	Winter wheat	**PL**
GW0279	Nateja	Winter wheat	**PL**
GW0280	Nutka	Winter wheat	**PL**
GW0281	Ostka Strzelecka	Winter wheat	**PL**
GW0282	Rywalka	Winter wheat	**PL**
GW0283	Rubens	Winter wheat	**PL**
GW0284	Satyna	Winter wheat	**PL**
GW0285	Smuga	Winter wheat	**PL**

GW0286	Sukces	Winter wheat	**PL**
GW0287	Tonacja	Winter wheat	**PL**
GW0288	Wydma	Winter wheat	**PL**
GW0289	Zawisza	Winter wheat	**PL**
GW0290	Zyta	Winter wheat	**PL**
GW0291	Aperitiv	Winter wheat	**S**
GW0292	Kosack	Winter wheat	**S**
GW0293	Lars	Winter wheat	**S**
GW0294	Marshal	Winter wheat	**S**
GW0295	Stava	Winter wheat	**S**
GW0296	SW Gnejs	Winter wheat	**S**
GW0297	SW Harnesk	Winter wheat	**S**
GW0298	SW Harpun	Winter wheat	**S**
GW0299	SW Hurtig	Winter wheat	**S**
GW0300	SW Skotte	Winter wheat	**S**
GW0301	Alchemy	Winter wheat	**UK**
GW0302	Ambrosia	Winter wheat	**UK**
GW0303	Battalion	Winter wheat	**UK**
GW0304	Brompton	Winter wheat	**UK**
GW0305	Claire	Winter wheat	**UK**
GW0306	Consort	Winter wheat	**UK**
GW0307	Cordiale	Winter wheat	**UK**
GW0308	CPBT W130	Winter wheat	**UK**
GW0309	Deben	Winter wheat	**UK**
GW0310	Duxford	Winter wheat	**UK**
GW0311	Einstein	Winter wheat	**UK**
GW0312	Gatsby	Winter wheat	**UK**
GW0313	Gladiator	Winter wheat	**UK**
GW0314	Glasgow	Winter wheat	**UK**
GW0315	Gulliver	Winter wheat	**UK**
GW0316	Humber	Winter wheat	**UK**
GW0317	Hyperion	Winter wheat	**UK**
GW0318	Istabraq	Winter wheat	**UK**
GW0319	Limerick	Winter wheat	**UK**
GW0320	Malacca	Winter wheat	**UK**
GW0321	Marksman	Winter wheat	**UK**
GW0322	Mascot	Winter wheat	**UK**
GW0323	Monty	Winter wheat	**UK**
GW0324	Musketeer	Winter wheat	**UK**
GW0325	Oakley	Winter wheat	**UK**

GW0326	Robigus	Winter wheat	**UK**
GW0327	Soissons	Winter wheat	**F**
GW0328	Solstice	Winter wheat	**UK**
GW0329	Timber	Winter wheat	**UK**
GW0330	Velocity	Winter wheat	**UK**
GW0331	Xi19.	Winter wheat	**UK**
GW0332	Zebedee	Winter wheat	**UK**
GW0333	Welford	Winter wheat	**UK**
GW0334	SW Tataros	Winter wheat	**NL**
GW0335	Vivant	Winter wheat	**NL**
GW0336	Apollo	Winter wheat	**D**
GW0337	Ares	Winter wheat	**D**
GW0338	Arina	Winter wheat	**CH**
GW0339	Caesar	Winter wheat	**F**
GW0341	Exsept	Winter wheat	**D**
GW0342	Hanseat	Winter wheat	**D**
GW0343	Haven	Winter wheat	**UK**
GW0344	Herzog	Winter wheat	**_**
GW0345	Hussar	Winter wheat	**UK**
GW0346	Ibis	Winter wheat	**D**
GW0347	Kanzler	Winter wheat	**D**
GW0348	Lynx	Winter wheat	**UK**
GW0349	Mikon	Winter wheat	**D**
GW0350	Orestis	Winter wheat	**D**
GW0351	Prinz	Winter wheat	**D**
GW0352	Ramiro	Winter wheat	**D**
GW0354	Renan	Winter wheat	**F**
GW0355	Savannah	Winter wheat	**UK**
GW0356	Slejpner	Winter wheat	**DK**
GW0357	Sperber	Winter wheat	**D**
GW0358	Travix	Winter wheat	**D**
GW0359	Urban	Winter wheat	**D**
GW0361	Viscount CPBT W 136	Winter wheat	**UK**
GW0363	Alceste	Winter wheat	**D**
GW0366	Monsun	Summer wheat	**D**
GW0367	Taifun	Summer wheat	**D**
GW0368	Marin	Summer wheat	**D**
GW0369	KWS Scirocco LP 509.3.04	Summer wheat	**D**
GW0370	KWS Chamsin LP 779.2.04	Summer wheat	**D**
GW0371	KWS Aurum LP 819.4.04	Summer wheat	**D**

GW0372	Vanek	Summer wheat	**PL**
GW0373	Trappe	Summer wheat	**PL**
GW0374	Bryza	Summer wheat	**PL**
GW0375	H05606	Summer wheat	**F**
GW0376	Baguette 11	Summer wheat	**F**
GW0377	P05311	Summer wheat	**F**
GW0378	P05312	Summer wheat	**F**
GW0379	P06079	Summer wheat	**F**
GW0380	Jafet	Winter wheat	**D**
GW0381	Profilius	Winter wheat	**D**
GW0382	Tabasco	Winter wheat	**D**
GW0383	Pamier	Winter wheat	**D**
GW0384	JB Asano	Winter wheat	**D**
GW0294a	Philius	Winter wheat	**D**
GW0385	Sophytra	Winter wheat	**D**

****Abbreviations for countries:**

D = Germany
F = France
PL = Poland
DK = Denmark
A = Austria
CZ = Czech Republic
UK = United Kingdom
S = Sweden
CH = Switzerland
NL = Netherlands

Table S2: Phenotypic variation for grain nutrients concentration [µg/g] among three years (2015/2016/2017) and BLUE.

Nutrient-Year	Maximum	Minimum	Mean
**Ca-2015**	797.2	334.6	497.3
**Ca-2016**	599.1	234.3	378.8
**Ca-2017**	597.8	208.5	314.2
**Ca-BLUEs**	590.7	261.6	396.9
**K-2015**	6431	3854	4936
**K-2016**	6727	3966	5153
**K-2017**	6019	3495	4660
**K-BLUEs**	5995	3995	4916
**Mg-2015**	1862	1007	1305
**Mg-2016**	1988	1015	1337
**Mg-2017**	1804	963.9	1284
**Mg-BLUEs**	1706	1063	1308
**Mn-2015**	49.22	23.37	32.29
**Mn-2016**	62.2	27.1	41.25
**Mn-2017**	48.98	24.67	32.40
**Mn-BLUEs**	51.93	25.43	35.31
**P-2015**	5043	2943	3784
**P-2016**	5807	3455	4462
**P-2017**	4809	2946	3761
**P-BLUEs**	4757	3459	4002
**S-2015**	2368	1390	1708
**S-2016**	1913	1157	1431
**S-2017**	1903	974.8	1251
**S-BLUEs**	1959	1206	1463

Table. S3: Analysis of variance (ANOVA) and heritability for nutrients concentration [µg/g] in European wheat panel (369 genotypes)**.**

Minerals	Variation source			H^2
	Genotype	Year	Genotype X Year	
**Ca**	***	**	ns	0.87
**K**	***	*	ns	0.79
**Mg**	***	*	ns	0.84
**Mn**	*	ns	ns	0.80
**P**	**	*	ns	0.72
**S**	***	**	ns	0.79

The degree of significance indicated as * $p \leq 0.05$, ** $P<0.01$, *** $P<0.001$; ns denotes not-significant.

Table S4-A: List of significant (–log10 (p) >3.0) marker trait associations of the SNP markers (90k and 35k arrays) in the wheat panel based on BLUE data.

Trait	SNP	Chromosome	Position_cM	Position_bp	_log p value	MAF*	Effect	R2
**Ca**	AX-94865782	1A	63.4	3.56E+08	4.16	0.080737	13.023249	2.369888
**Ca**	wsnp_Ex_c22284_31478675	1A	34.8	5.14E+08	3.11	0.050992	15.41533644	1.768251
**Ca**	BS00022176_51	1B	75.6	6.37E+08	4.50	0.467422	-7.84540666	0.166212
**Ca**	AX-94997333	1D	24	18075042	3.31	0.134561	-10.250629	1.173049
**Ca**	BS00049644_51	2A	66.6	6.05E+08	8.97	0.451841	-13.6641341	2.616769
**Ca**	BS00035335_51	2B	78.8	5.92E+08	4.56	0.137394	-11.8299262	1.418346
**Ca**	Kukri_c900_1334	2B	144.2	7.97E+08	3.09	0.056657	-15.2181632	0.638508
**Ca**	wsnp_BG275030D_Ta_2_2	2D	107.2	35504582	3.03	0.072238	12.85373615	0.306905
**Ca**	Excalibur_rep_c71645_94	3B	5.4	3605996	5.64	0.307365	-8.69866977	2.34428
**Ca**	RAC875_c8642_231	5A	114.5	6985100	10.89	0.087819	26.39704101	10.5404
**Ca**	AX-94644169	5B	103.2	5.8E+08	4.88	0.279037	9.449613484	3.317796
**Ca**	AX-94424011	6B	93.3	6.96E+08	3.54	0.133144	-9.3881254	0.460713
**Ca**	BS00033442_51	7A	87.2	1.26E+08	5.50	0.436261	8.428977478	1.008175
**K**	AX-95144468	1B	40.5	1.81E+08	7.82	0.155125	103.8115434	1.887891
**K**	AX-94642361	2B	68.3	1.92E+08	3.90	0.048476	108.0951809	1.575342
**K**	BS00057445_51	3A	149.9	25388020	4.08	0.358726	47.21455547	0.981617
**K**	AX-95199446	3B	133.9488	8.15E+08	9.32	0.077562	158.7322521	3.007818
**K**	AX-94518705	4A	111.9	6.28E+08	4.29	0.048476	100.8888593	1.034287

**K**	BS00100738_51	4B	5.4	6.61E+08	4.68	0.102493	-81.3234559	1.179582
**K**	RAC875_c8642_231	5A	114.5	6.99E+08	6.65	0.088643	-117.04167	3.406486
**K**	Kukri_c62247_248	5B	115.9	4.86E+08	6.25	0.038781	165.7875729	2.301398
**K**	AX-94814333	6B	48.3	1.56E+08	3.89	0.452909	39.69401314	1.318037
**K**	BS00040601_51	7A	88.8	1.02E+08	7.18	0.076177	126.4298514	2.171542
**K**	RAC875_c1962_1517	7B	65.4	2.08E+08	5.27	0.037396	138.4556633	1.609515
**K**	AX-94402434	7D	111.5	4.15E+08	3.13	0.121884	61.76202289	0.490449
**K**	AX-94523269	7D	88.5	1.02E+08	3.69	0.059557	-85.9720998	0.907903
**K**	AX-94589433	7D	195.1	6.31E+08	3.30	0.239612	53.21333224	1.29721
**K**	RAC875_rep_c110526_324	7D	4.9	6.31E+08	3.56	0.240997	55.63446766	1.29721
**Mg**	Excalibur_rep_c69522_83	1B	111.7	6.86E+08	4.13	0.350416	-12.113623	1.74411
**Mg**	wsnp_CAP7_c402_217331	2B	64.3	1.42E+08	3.10	0.188366	-17.2263018	1.482287
**Mg**	Tdurum_contig100787_79	3B	130.3	8.11E+08	3.30	0.117729	20.62807382	0.403514
**Mg**	wsnp_JD_c30422_23944042	3B	44.9	1.01E+08	4.30	0.16205	-18.6697961	1.598614
**Mg**	AX-95652024	4A	120.4	6.42E+08	3.05	0.290859	14.26748894	1.198364
**Mg**	AX-94914669	4B	66.4	5.79E+08	3.14	0.094183	-110.541606	0.682344
**Mg**	RAC875_c62816_54	4B	40.6	5.9E+08	6.10	0.090028	-33.2191731	1.515113
**Mg**	RAC875_c8642_231	5A	114.5	6.99E+08	9.15	0.088643	49.3724021	6.570732
**Mg**	AX-94505827	5B	117.2	6.04E+08	4.53	0.5	15.27157668	1.490212
**Mg**	AX-94972025	5B	125.8	6.34E+08	5.45	0.203601	-24.4061228	1.151098

**Mg**	AX-94454656	5D	13.9	29505617	3.28	0.33518	14.28661818	1.097574
**Mg**	RAC875_c52504_215	6A	34.1	18704862	4.63	0.232687	20.81613131	1.241381
**Mg**	AX-95105594	7A	129.7	6.89E+08	4.09	0.134349	-21.5195649	0.411828
**Mg**	BS00053287_51	7B	63.9	4.91E+08	5.01	0.163435	24.45845947	1.441883
**Mn**	AX-95230131	2B	80.4	6.38E+08	3.12	0.114958	-0.78361657	1.317396
**Mn**	RFL_Contig5682_86	3B	55.1	5.1E+08	4.32	0.419668	-0.63130014	1.586139
**Mn**	Kukri_c17900_887	4B	71.8	17367824	7.41	0.243767	0.920606948	2.552296
**Mn**	RAC875_c8642_231	5A	114.5	6.99E+08	10.26	0.088643	1.837677787	7.597879
**Mn**	Tdurum_contig48760_112	5A	27	5.36E+08	3.10	0.272853	0.652091433	0.93985
**Mn**	BS00063769_51	5B	140	47586810	5.21	0.245152	-0.70249541	1.935615
**Mn**	RAC875_c28831_558	5B	169.5	12326585	3.22	0.160665	0.714812353	0.183781
**Mn**	wsnp_Ex_c2459_4591587	5B	169.5	12324513	3.17	0.16205	-0.70397606	0.167242
**Mn**	AX-95089337	6B	49	2.03E+08	3.36	0.437673	0.301959621	0.882142
**Mn**	RAC875_c34939_86	7B	142	7.42E+08	3.91	0.221607	0.554146165	1.488372
**P**	BS00022220_51	1A	36.4	5.09E+08	3.61	0.437673	-29.080593	2.143028
**P**	AX-94400797	2A	64.3	72931314	4.69	0.247922	44.51955472	2.423501
**P**	AX-95165048	2A	64.3	77941594	3.02	0.144044	49.45185155	0.76855
**P**	Kukri_rep_c94009_116	2B	64.3	1.42E+08	4.77	0.216066	-48.931118	2.012432
**P**	AX-94387711	3A	56.8	4.33E+08	4.54	0.157895	53.95610579	1.189539
**P**	wsnp_Ex_c8360_14085858	3B	6.9	5953263	3.03	0.105263	42.49792043	0.530758

**P**	wsnp_JD_c30422_23944042	3B	44.9	1.01E+08	3.93	0.16205	-44.9864527	1.364756
**P**	AX-95652024	4A	120.4	6.42E+08	5.03	0.290859	46.94773649	3.234594
**P**	Ku_c48056_436	4B	40.6	5.78E+08	3.81	0.15374	44.87892137	1.545333
**P**	CAP8_c9110_427	4D	57.2	1.21E+08	5.66	0.33241	-50.135602	3.176519
**P**	AX-94487713	5A	84.9	6.57E+08	3.43	0.264543	34.71300128	2.452774
**P**	AX-94474369	5B	166.5	6.94E+08	4.13	0.185596	-46.3825587	1.716247
**P**	RFL_Contig5314_1147	6A	86.1	5.85E+08	3.69	0.300554	-36.3282501	2.685148
**P**	AX-94670702	6B	1	1970037	3.13	0.445983	-32.3831089	2.111952
**P**	AX-95167797	7B	60.6	1.29E+08	5.55	0.1759	62.07673021	0.905962
**S**	BS00088035_51	1A	35.6	5.13E+08	3.26	0.455679	11.38620222	0.892565
**S**	wsnp_Ra_c16080_24638622	1A	41.9	4.88E+08	3.84	0.293629	16.3541083	0.042194
**S**	RFL_Contig5906_694	1B	15.7	16217136	3.18	0.189751	13.07539657	1.259046
**S**	Tdurum_contig47550_699	1B	56.4	5.67E+08	3.68	0.364266	12.55272015	2.163067
**S**	AX-94804916	2B	64.3	1.42E+08	3.09	0.306094	-23.4812646	0.181286
**S**	BS00022417_51	2B	64.3	1.42E+08	3.07	0.304709	23.4743138	0.225334
**S**	Excalibur_c15031_73	2B	64.3	1.39E+08	5.95	0.249307	-18.2713479	2.014289
**S**	GENE-3867_1133	2B	64.3	1.82E+08	3.08	0.306094	-23.4618006	0.259539
**S**	RAC875_c15649_1101	2B	64.3	1.81E+08	3.01	0.303324	23.10803458	0.221808
**S**	RAC875_c41242_133	2B	64.3	1.42E+08	3.05	0.304709	23.26165927	0.216161
**S**	RAC875_rep_c110753_119	2D	111.1	31948344	4.33	0.067867	29.99233661	1.293501

**S**	AX-94922974	3B	58.2135	5.8E+08	3.29	0.454294	12.28836128	0.957701
**S**	AX-94464372	4A	128.2	7.1E+08	4.82	0.126039	-21.224431	1.037874
**S**	RAC875_c39226_131	4B	43.7	5.6E+08	4.00	0.17867	-14.8126113	2.240975
**S**	RAC875_c8642_231	5A	114.5	6.99E+08	4.63	0.088643	28.69480662	3.714203
**S**	Excalibur_c38433_291	5B	78.7	5.71E+08	4.94	0.461219	-18.271148	1.662281
**S**	BS00022951_51	6A	34.1	24332134	4.16	0.34349	-14.5186506	0.925341
**S**	Tdurum_contig22364_380	7A	77.7	6.11E+08	3.14	0.126039	14.77201456	1.014281
**S**	AX-94680840	7B	1	430216	3.12	0.429363	-12.1516571	1.570207

***MAF=minor allele frequency**

Table S4-B: List of significant (–log10 (p) >3.0) marker trait associations of the SNP markers (90k and 35k arrays) above the bonferroni correction (=5.42) for the wheat panel based on BLUE data.

Trait	SNP	Chromosome#	Chromosome	Position_cM	Position_bp	P.value	log	maf	effect	R2
**Ca**	BS00049644_51	4	2A	66.6	605184949	1.06E-09	8.9737	0.4518	-13.6641341	2.6168
**Ca**	Excalibur_rep_c71645_94	8	3B	5.4	3605996	2.30E-06	5.6384	0.3074	-8.69866977	2.3443
**Ca**	RAC875_c8642_231	13	5A	114.5	698510016	1.30E-11	10.887	0.0878	26.39704101	10.54
**Ca**	BS00033442_51	19	7A	87.2	125715708	3.16E-06	5.4999	0.4363	8.428977478	1.0082
**K**	AX-95144468	2	1B	40.5	180678022	1.51E-08	7.8221	0.1551	103.8115434	1.8879
**K**	AX-95199446	8	3B	133.95	815479837	4.81E-10	9.3175	0.0776	158.7322521	3.0078
**K**	RAC875_c8642_231	13	5A	114.5	6985100.2	2.22E-07	6.6545	0.0886	-117.04167	3.4065
**K**	Kukri_c62247_248	14	5B	115.9	485997910	5.58E-07	6.2534	0.0388	165.7875729	2.3014
**K**	BS00040601_51	19	7A	88.8	102058807	6.60E-08	7.1807	0.0762	126.4298514	2.1715
**Mg**	RAC875_c62816_54	11	4B	40.6	578031318	8.04E-07	6.095	0.09	-33.2191731	1.5151
**Mg**	RAC875_c8642_231	13	5A	114.5	698510016	7.08E-10	9.1499	0.0886	49.3724021	6.5707
**Mg**	AX-94972025	14	5B	125.8	634279894	3.55E-06	5.4497	0.2036	-24.4061228	1.1511
**Mn**	Kukri_c17900_887	11	4B	71.8	17367824	3.87E-08	7.4123	0.2438	0.920606948	2.5523
**Mn**	RAC875_c8642_231	13	5A	114.5	698510016	5.45E-11	10.264	0.0886	1.837677787	7.5979
**P**	CAP8_c9110_427	12	4D	57.2	121181674	2.20E-06	5.6567	0.3324	-50.135602	3.1765
**P**	AX-95167797	20	7B	60.6	128810384	2.79E-06	5.5541	0.1759	62.07673021	0.906
**S**	Excalibur_c15031_73	5	2B	64.3	139068437	1.12E-06	5.9492	0.2493	-18.2713479	2.0143

Table S5: Allelic variation analysis output for RAC875_c8642_231 marker underlying nutrient minerals (μg.g^{-1}) accumulation in wheat grains.

Genotype	RAC875_c8642_231	Ca	Mg	Mn	P	S	K	Fe	Zn	TKW (g)
**GW0001**	C	378.5	1399	33.63	3917	1357	5212	48.25	40.22	55.20
**GW0002**	C	334.4	1310	34.84	3892	1396	5099	36.93	34.56	61.92
**GW0003**	C	387	1408	37.91	4229	1500	4841	41.43	39.45	52.20
**GW0004**	C	342.3	1579	38.94	4513	1577	4743	43.71	41.88	47.65
**GW0005**	C	358.4	1615	37.98	4540	1750	5169	39.94	37.36	46.67
**GW0006**	C	318	1263	30.86	3804	1299	5196	30.34	29.87	53.60
**GW0007**	C	398.3	1322	35.35	3895	1405	4787	37.25	31.47	54.12
**GW0008**	C	385.9	1327	34.08	3985	1404	4619	37.82	34.4	52.35
**GW0009**	C	498.4	1413	37.6	4017	1486	4762	35.81	34.78	50.78
**GW0010**	C	406	1305	37.63	3886	1417	4781	34.62	33.15	54.00
**GW0011**	C	378.5	1244	35.4	3695	1350	4886	32.62	31.3	56.20
**GW0012**	C	427.3	1303	34.7	3936	1377	5065	32.98	34.67	53.53
**GW0013**	C	419.4	1346	35.3	4039	1374	5138	33.52	34.02	51.96
**GW0014**	C	406.9	1340	37.81	4081	1441	5054	38.59	38.47	48.20
**GW0015**	C	469.1	1396	41.67	4142	1524	4572	34.17	37.86	51.40
**GW0016**	C	329.7	1437	36.68	3990	1358	5129	33.41	36.17	59.00
**GW0017**	C	382.9	1391	34.76	4274	1425	5501	37.55	36.15	51.37
**GW0019**	C	323.9	1326	35.33	4087	1589	5071	31.91	35.17	57.40
**GW0021**	C	418.4	1430	41.8	4168	1485	5032	37.86	35.67	59.60
**GW0022**	C	407.6	1295	33.63	3719	1394	4666	29.35	28.17	56.08
**GW0023**	C	325.2	1399	35.38	4277	1743	4668	34.61	37.53	58.80
**GW0024**	C	362.6	1440	35.2	4113	1506	4914	31.86	33	53.14
**GW0025**	C	390.3	1324	34.85	4148	1412	5041	29.27	33.37	46.80

**GW0026**	C	316.6	1309	35.64	3813	1330	5334	32.08	28.98	46.60
**GW0027**	C	375.7	1360	35.23	3903	1332	5441	30.61	32.06	50.80
**GW0028**	C	332.3	1380	35.31	4121	1384	4593	30.35	32.91	46.60
**GW0029**	C	371	1386	35.55	4059	1377	4989	31.62	31.89	48.82
**GW0030**	C	365.4	1221	33.16	3866	1379	5257	31.84	32.09	52.75
**GW0031**	C	352.8	1251	36.53	3866	1316	5266	31.09	31.74	50.20
**GW0032**	C	416.8	1383	38.63	4141	1361	4670	36.62	36.22	51.20
**GW0033**	C	419.4	1316	36.69	3996	1459	5161	39.11	40.75	42.55
**GW0034**	C	357	1266	36.43	4026	1436	5480	39.4	39.22	56.27
**GW0035**	C	348	1275	38.11	3889	1395	5309	35.94	37.3	57.00
**GW0036**	C	Na	1261	33.59	3870	1435	5217	38.13	34.04	58.20
**GW0037**	C	376.8	1233	36.61	3887	1343	4705	33.31	30.21	59.40
**GW0038**	C	298.6	1382	34.17	4201	1450	4726	35.63	35.83	53.06
**GW0039**	C	298.7	1324	32.73	4035	1341	5258	32.52	33.87	55.40
**GW0040**	C	364.5	1258	33.64	4146	1504	5076	41.68	42.42	56.60
**GW0041**	C	390.3	1334	37.78	4057	1343	4881	34.67	35.48	54.00
**GW0042**	C	360	1340	32.17	4102	1368	5189	37.28	34.64	55.80
**GW0043**	C	399.6	1247	35.06	3881	1327	4833	32.53	34.79	47.60
**GW0044**	C	362.4	1340	36.57	3988	1420	5247	39.7	35.83	46.08
**GW0045**	C	322.4	1441	36.36	4226	1562	4533	38.28	36.44	48.60
**GW0046**	C	321.3	1251	32.21	3865	1490	4634	35.73	31.48	52.60
**GW0047**	C	350.7	1238	34.58	3865	1444	4431	33.37	31.29	57.00
**GW0048**	C	368.5	1356	34.62	4274	1504	5145	36.57	38.29	56.67
**GW0049**	C	358.7	1413	37.74	4342	1456	4805	40.55	38.02	57.60
**GW0050**	C	341.7	1390	40.68	4022	1459	4578	43.61	37.49	52.20
**GW0051**	C	361.2	1262	36.8	3821	1432	4585	35.42	33.51	52.00

**GW0052**	C	374.1	1280	36.22	3802	1440	5004	32.65	30.96	51.80
**GW0053**	C	420	1213	35.82	3797	1262	5098	31.17	34.33	53.40
**GW0054**	C	424	1385	41.2	4381	1463	5240	36.65	38.96	47.60
**GW0055**	C	339.9	1372	41.38	4086	1426	4888	37.21	37.52	51.40
**GW0056**	C	445.3	1305	37.45	3980	1479	5156	30.05	31.24	56.79
**GW0057**	C	378.7	1244	36.45	4195	1602	5051	34.39	33.66	49.80
**GW0058**	C	457.8	1304	34.96	4086	1432	5048	28.82	34.75	47.20
**GW0059**	C	378	1439	35.12	4160	1537	4488	33.1	37.85	56.80
**GW0060**	C	362.1	1331	30.6	3999	1479	5278	32.6	32.88	46.80
**GW0061**	C	402.9	1441	35.11	4441	1583	4422	37.79	44.23	52.20
**GW0062**	C	374.5	1371	36.37	4042	1429	4620	31.81	33.39	51.40
**GW0064**	C	389.9	1496	38.73	4675	1517	5457	36.12	40.78	47.60
**GW0065**	C	347	1291	33.31	3809	1462	4353	34.52	32.53	45.80
**GW0066**	C	339.5	1193	33.71	3690	1461	4600	30.98	30.46	56.80
**GW0067**	C	403.4	1300	33.31	4098	1485	5251	38.97	36.78	64.00
**GW0068**	C	384	1293	35.27	4172	1562	4782	34.84	36.6	55.49
**GW0069**	C	388.7	1308	34.09	3952	1402	4820	32.22	34.15	46.40
**GW0070**	C	407.8	1408	36.26	4105	1620	4845	32.61	35.17	50.40
**GW0071**	C	402.2	1229	35.44	4007	1498	4871	32.03	35.18	53.60
**GW0072**	C	443.3	1415	41.91	4368	1571	5116	41.83	39.56	49.80
**GW0073**	C	335.6	1286	34.79	3995	1522	4820	31.32	31.25	56.80
**GW0074**	C	441.1	1263	37.93	4169	1523	5169	34.6	33.69	56.54
**GW0075**	C	330.6	1320	31.96	4006	1328	5241	30.78	30.72	58.98
**GW0076**	C	323.5	1272	35.45	4067	1491	4723	36.21	35.5	50.00
**GW0077**	C	360.5	1245	34.09	3681	1468	4744	34.78	31.13	53.20
**GW0078**	C	343.9	1224	30.69	3529	1301	4439	33.61	29.7	45.80

**GW0079**	C	364.9	1329	34.88	3684	1335	4548	34.78	32.21	56.20
**GW0080**	C	285.3	1234	34.32	3842	1519	5072	36.82	33.47	49.40
**GW0081**	C	399.2	1374	35.32	4284	1408	4893	35.42	36.4	53.40
**GW0082**	C	371	1269	37.87	4031	1315	5141	32.05	36.97	45.00
**GW0083**	C	417	1442	40.99	4333	1509	5099	38.22	43.17	52.40
**GW0084**	C	305.1	1383	34.64	4316	1546	4852	36.3	36.99	47.00
**GW0085**	C	316.4	1148	32.92	3787	1386	4303	33.35	35.3	46.80
**GW0086**	C	358.9	1317	36.65	3956	1536	4776	39.03	35.62	46.40
**GW0087**	C	328.4	1133	29.69	3762	1323	4602	29.24	27.6	50.20
**GW0088**	C	261.6	1136	29.95	3804	1418	4626	36.39	33.35	48.60
**GW0089**	C	368	1166	31.9	3827	1424	4253	30.54	31.35	46.40
**GW0090**	C	391	1375	33.75	4259	1467	4999	33.34	32.61	50.40
**GW0091**	C	425.2	1223	29.56	3694	1353	5106	32	27.52	54.20
**GW0092**	C	370.6	1252	34.29	4115	1362	5029	28.4	29.99	48.63
**GW0093**	C	408.5	1172	31.02	3780	1320	4937	29.05	30.3	45.60
**GW0094**	C	312.1	1219	29.96	3999	1520	4980	30.04	31.65	53.00
**GW0095**	C	364.1	1250	32.75	3980	1454	4842	35.32	31.75	52.00
**GW0096**	C	355.1	1316	34.6	4069	1474	4506	31.14	31.33	49.80
**GW0097**	C	322.7	1223	31.13	4019	1513	5139	33.06	32.54	52.80
**GW0098**	C	399.9	1231	32.96	3983	1413	5288	31.83	29.59	55.51
**GW0099**	C	417.4	1260	31.69	3459	1445	4410	28.2	25.05	48.40
**GW0100**	C	380.7	1271	38.28	3663	1315	4952	31.61	28.52	49.80
**GW0101**	C	436.2	1378	37.54	4082	1489	4566	31.82	32.82	53.78
**GW0102**	C	444.3	1201	34.44	3921	1439	4400	32.42	32.9	48.40
**GW0103**	C	360.9	1236	32.66	3733	1402	4683	29.8	34.73	52.78
**GW0104**	C	335.1	1357	39.95	4059	1444	4216	41.02	39.47	60.59

**GW0106**	C	367.6	1256	32.53	3975	1393	4961	31.03	30.67	57.40
**GW0107**	C	449.3	1229	35.02	3922	1552	5018	37.25	35	49.80
**GW0109**	C	364.1	1249	33.82	3865	1345	4941	31.46	29.84	49.41
**GW0110**	C	Na	1094	30.26	3794	1206	5035	31.09	29.06	Na
**GW0111**	C	449.7	1199	35.46	3770	1427	4786	30.6	29.57	52.40
**GW0113**	C	464.5	1321	33.68	4199	1395	5690	29.25	33.48	52.20
**GW0114**	C	366.5	1329	32.36	4083	1468	4988	30.74	32.63	60.00
**GW0115**	C	438.3	1131	35.36	3710	1338	5243	33.99	30.98	49.25
**GW0116**	C	401.8	1327	33.34	4128	1461	5212	33.42	32.18	51.22
**GW0117**	C	373	1419	32.87	4235	1585	4817	38.07	33.58	49.61
**GW0118**	C	410.4	1233	31.94	3978	1504	5027	31.62	30.86	48.85
**GW0120**	C	377.2	1289	35.71	4158	1460	4653	33.75	33.24	50.98
**GW0121**	C	402.5	1245	31.3	4033	1319	5183	30.33	28.86	47.60
**GW0122**	C	358	1170	36.39	4019	1394	4856	30.04	29.89	51.54
**GW0123**	C	373.2	1414	36.76	4265	1475	4694	35.83	36.09	52.40
**GW0124**	C	352	1115	29.02	3728	1347	4793	30.19	31.95	51.96
**GW0125**	C	412.4	1186	34.23	3870	1491	4609	32.69	31.21	52.88
**GW0126**	C	Na	1125	33.57	3736	1562	4620	34.13	34.19	55.77
**GW0127**	C	364.2	1229	31.69	3997	1415	5077	35.42	30.24	51.04
**GW0128**	C	374.6	1149	31.44	4006	1372	4968	34.51	32.39	58.68
**GW0130**	C	403.9	1091	30.43	3872	1417	4718	34.42	31.21	58.08
**GW0131**	C	452.9	1289	35.4	4290	1591	5140	36.85	35.13	53.60
**GW0132**	C	386.2	1103	29.73	3796	1429	5166	28.48	30.86	48.82
**GW0133**	C	400.4	1353	31.95	3888	1352	4567	30.46	29.98	45.19
**GW0134**	C	299.7	1266	32.14	4134	1330	4855	32.98	30.97	56.47
**GW0135**	C	453.6	1181	37.81	3933	1377	4779	29	29.24	57.84

**GW0136**	C	399.5	1063	31.58	3477	1303	4588	24.58	26.14	46.60
**GW0137**	C	372.2	1299	37.06	3947	1263	4780	32.6	32.84	55.80
**GW0138**	C	387	1153	32.34	3744	1408	4807	28.29	28.75	48.63
**GW0140**	C	336.4	1180	33.31	3859	1383	4444	31.84	31.7	58.60
**GW0142**	C	395.6	1141	36.19	4014	1377	4743	28.13	29.5	46.60
**GW0143**	C	Na	Na	Na	Na	Na	Na	46.47	33.38	Na
**GW0144**	C	421.4	1156	33.89	3938	1374	4987	29.73	28.83	49.02
**GW0146**	C	442	1207	37.97	4043	1431	4862	30.32	30.26	49.60
**GW0147**	C	388.9	1067	29	3529	1316	4337	27.92	31.61	60.00
**GW0148**	C	438.2	1231	36.17	3870	1422	4726	37.04	34.7	55.49
**GW0149**	C	345.7	1288	31.67	4134	1399	4767	31.12	31.9	42.50
**GW0150**	C	416.4	1308	34.18	4135	1484	5127	33.89	34	49.80
**GW0151**	C	467.5	1318	39.91	4441	1626	5532	46.07	41.39	47.35
**GW0152**	C	412.9	1102	32.25	3951	1328	4971	24.42	27.23	47.12
**GW0153**	C	402.1	1247	37.6	4052	1447	4946	30.19	33.52	54.20
**GW0155**	C	514.2	1346	40.94	4041	1568	4540	34.99	36.88	51.37
**GW0157**	C	445.1	1336	37.18	4008	1573	4803	36.15	37.03	50.74
**GW0158**	C	400.3	1355	36.46	4257	1498	5348	39.02	40.31	60.00
**GW0159**	C	406.9	1282	31.09	4160	1412	5068	30.56	33.29	55.00
**GW0161**	C	363.6	1185	33.18	3783	1488	4919	40.54	37.63	60.00
**GW0162**	C	450.9	1386	33.75	4682	1581	4971	39.78	42.77	50.00
**GW0164**	C	422.9	1199	35.16	4063	1385	5169	34.91	35.28	57.96
**GW0165**	C	443.9	1305	40.59	4496	1601	5081	40.2	42.77	60.19
**GW0166**	C	379.1	1177	33.2	3999	1348	4867	33.17	37.5	48.85
**GW0167**	C	459.5	1193	35.49	4248	1554	4885	33.83	39.27	62.00
**GW0170**	C	403.9	1258	36.18	4218	1425	5203	35.19	32.04	50.20

**GW0171**	C	390.4	1304	34.45	3982	1480	5140	33.16	34.8	60.75
**GW0172**	C	409.4	1202	32.67	3927	1362	4849	29.38	29.99	52.00
**GW0173**	C	392.7	1432	34.45	4018	1469	4597	33	34.64	49.61
**GW0174**	C	376.4	1315	32.32	4111	1483	4603	34.42	39.38	50.59
**GW0175**	C	436.7	1301	33.66	4148	1460	5129	32.94	31.58	52.20
**GW0176**	C	461.7	1284	36.62	4171	1541	4892	33.91	32.61	46.67
**GW0177**	C	350.9	1380	32.15	4071	1324	4454	32.38	29.19	48.40
**GW0178**	C	346.5	1210	32.77	3742	1388	4676	31.43	32.01	47.20
**GW0180**	C	426.7	1204	36.57	3969	1383	4932	27.66	29.67	47.06
**GW0181**	C	338.8	1299	36.57	4203	1457	5139	36.24	35.5	52.75
**GW0182**	C	397.2	1124	29.82	3558	1368	4739	29.77	28.92	51.00
**GW0183**	C	501.5	1337	37.26	4021	1492	4519	36.5	32.62	57.20
**GW0184**	C	476.6	1131	32.48	4036	1464	4534	30.77	33.32	52.94
**GW0185**	C	392.6	1185	33.69	3972	1365	4871	27.64	27.11	48.65
**GW0186**	C	431.5	1294	33.62	4184	1444	5037	28.84	33.08	51.76
**GW0187**	C	Na	1156	32.25	3699	1311	4998	27.78	34.08	52.69
**GW0188**	C	383.9	1265	33.45	4046	1367	5085	31.38	33.87	51.37
**GW0189**	C	394.7	1378	47.21	4439	1619	4158	46.1	42.58	50.59
**GW0190**	C	483.5	1246	34.92	4048	1456	5132	31.89	33.13	57.45
**GW0191**	C	426.6	1288	34.51	4054	1408	4686	29.41	30.53	47.00
**GW0192**	C	387.1	1156	33.79	3766	1451	4660	31.12	31.4	49.80
**GW0193**	C	376.8	1162	33.53	4080	1457	5130	29.07	32.19	52.12
**GW0194**	C	461.6	1183	36.61	3908	1492	5003	30.25	34.25	56.53
**GW0195**	C	Na	Na	Na	Na	Na	Na	37.18	38.36	Na
**GW0196**	C	421.2	1214	34.48	3865	1443	4767	31.03	31.12	57.36
**GW0197**	C	394.4	1469	42.49	4523	1664	4199	45.75	48.45	53.73

**GW0198**	C	400.3	1230	34.07	3987	1463	4962	35.87	43.09	59.20
**GW0199**	C	360	1358	37.85	4409	1649	4526	35.15	38.25	53.53
**GW0200**	C	513.5	1297	25.43	4210	1279	5134	31.14	31.13	53.40
**GW0201**	C	419.9	1134	36.3	3584	1444	4134	32	33.09	53.46
**GW0202**	C	357.3	1110	33.62	4031	1365	4970	32.54	31.82	56.80
**GW0204**	C	349.2	1287	36.61	4162	1473	5269	37.06	37.71	54.62
**GW0205**	C	407.9	1135	32.51	3698	1457	4522	31.64	34.03	47.88
**GW0206**	C	349.7	1133	32.68	3830	1547	4646	34.99	32.74	52.35
**GW0207**	C	409.4	1359	37.08	4223	1561	4803	39.14	36.72	56.33
**GW0210**	C	294.6	1146	28.42	3614	1284	5376	33.35	32.5	59.80
**GW0211**	C	346.1	1383	30.61	3831	1361	4772	34.05	37.25	47.45
**GW0212**	C	492.1	1357	37.32	3686	1290	4834	34.84	34.36	50.96
**GW0213**	C	399.8	1420	36.39	4113	1432	5355	37.99	36.1	53.53
**GW0214**	C	409.3	1419	30.63	3979	1404	5114	31.62	36.39	43.85
**GW0215**	C	Na	1523	41.78	4207	1586	4531	Na	34.46	Na
**GW0218**	C	385	1200	31.89	3863	1414	4832	29.05	32.2	60.60
**GW0220**	C	404.1	1362	39.69	3815	1435	4677	41.57	34.74	48.85
**GW0222**	C	368.5	1276	34.46	3845	1448	5129	34.61	35.76	45.29
**GW0223**	C	379.4	1285	33.09	3676	1393	5184	36.8	36.32	53.65
**GW0224**	C	334.7	1266	31.12	3619	1377	4496	29.33	31.09	47.45
**GW0225**	C	343.5	1195	30.93	3719	1356	5459	36.23	31.11	42.16
**GW0226**	C	330.5	1114	30.45	3576	1412	5170	30.53	30.29	51.96
**GW0228**	C	371.9	1158	34.77	3728	1327	5015	32.35	34.37	50.98
**GW0229**	C	354.8	1273	36.22	3904	1419	5022	40.2	38.8	55.64
**GW0230**	C	383.1	1285	35.47	4068	1310	5119	29.52	35.09	56.54
**GW0231**	C	379.7	1214	30.77	3733	1376	5358	33.97	34.67	58.85

**GW0232**	C	427.3	1167	35.32	3798	1347	5309	30.48	34.32	52.50
**GW0233**	C	389.4	1186	33.45	3534	1290	5002	28.38	31.98	56.23
**GW0234**	C	492	1257	33.41	3933	1583	4911	38.57	37.81	53.20
**GW0235**	C	409.6	1266	33.91	3570	1431	4893	32.06	31.28	57.45
**GW0236**	C	377.8	1252	36.56	4039	1442	5547	34.85	33.48	49.60
**GW0237**	C	377.9	1178	35.09	3546	1382	4907	31.91	31.07	49.22
**GW0238**	C	407.8	1188	35.78	3899	1383	5320	32.1	31.06	51.20
**GW0239**	C	352.5	1257	37.56	3735	1431	5043	36.27	35.51	53.53
**GW0240**	C	420.4	1153	34.38	3741	1404	5073	31.99	29.87	51.18
**GW0241**	C	360.1	1379	40.68	3889	1518	5136	38.07	35.87	58.20
**GW0242**	C	Na	1364	31.29	3893	1441	5521	Na	31.92	Na
**GW0243**	C	393.8	1310	31.15	3849	1395	5113	33.05	30.43	45.96
**GW0244**	C	341.6	1462	38.34	4231	1516	4443	32.8	35.72	55.40
**GW0249**	C	390.8	1493	35.26	4132	1572	4697	38.82	37.63	48.11
**GW0250**	C	316.9	1416	38.71	4119	1583	4866	39.95	41.44	52.16
**GW0252**	C	343.8	1207	31.99	3853	1401	4616	32.55	32.6	47.25
**GW0253**	C	373.3	1474	37.1	4320	1525	4875	38.26	44.28	57.20
**GW0254**	C	383.4	1436	39.73	4221	1553	4985	40.1	43.88	52.16
**GW0255**	C	406.9	1430	37.85	4140	1431	5279	38.99	42.46	59.61
**GW0256**	C	389.2	1414	36.88	4076	1409	5167	34.86	34.45	55.58
**GW0258**	C	401.2	1309	35.92	4357	1605	5650	39.2	39.25	55.19
**GW0259**	C	409.8	1234	36.58	3982	1447	5344	36	34.52	56.27
**GW0260**	C	375.2	1298	34.08	3813	1350	5122	31.66	30.3	53.58
**GW0262**	C	359.7	1450	39.34	3800	1476	5079	38.9	33.91	50.39
**GW0263**	C	313.3	1276	36.34	3826	1431	4930	33.9	32.78	53.92
**GW0264**	C	450.7	1358	35.81	3978	1579	4204	32.99	36.32	49.60

**GW0265**	C	334.2	1335	36.5	4061	1513	4778	34.13	34.55	60.00
**GW0266**	C	344.6	1290	37.3	3680	1410	4279	32.2	36.48	50.40
**GW0267**	C	433.2	1434	34.35	4194	1545	4508	34.23	38.7	51.20
**GW0268**	C	442.2	1566	43.68	4549	1647	4901	41.33	42.89	47.12
**GW0269**	C	498	1289	39.13	4103	1564	4774	39.04	44.42	56.67
**GW0270**	C	Na	1255	32.96	3670	1288	4748	29.77	26.66	56.00
**GW0271**	C	Na	Na	Na	Na	Na	Na	30.08	34.59	Na
**GW0272**	C	361.2	1351	38.91	4086	1556	4925	38.05	39.04	47.00
**GW0273**	C	430.4	1395	40.86	4263	1591	4817	37.87	36.63	52.80
**GW0274**	C	457.3	1430	43.15	4130	1583	5388	33.67	40.02	53.60
**GW0276**	C	558.3	1507	42.26	4236	1646	4623	41.47	40.9	51.00
**GW0277**	C	468.3	1402	32.88	4296	1512	5180	34.09	39.62	52.40
**GW0278**	C	445.1	1268	33.92	3903	1577	4761	31.37	34.75	56.12
**GW0279**	C	449.3	1460	38.72	4120	1570	4727	32.57	37.91	55.77
**GW0280**	C	455.6	1397	36.79	4066	1468	5117	37.24	41.46	51.54
**GW0282**	C	351.9	1391	36.03	4109	1474	4720	40.47	35.92	53.92
**GW0283**	C	475.3	1520	37.96	4471	1508	4881	32.71	38.79	40.60
**GW0284**	C	394.9	1359	40.84	4207	1482	5071	36.78	40.35	50.38
**GW0285**	C	339.3	1313	37.03	3993	1514	4484	36.24	37.23	51.37
**GW0286**	C	375.2	1301	36.52	4000	1494	4762	40.28	36.89	55.49
**GW0287**	C	364.6	1401	39.89	4099	1535	4671	39.87	42.6	55.29
**GW0288**	C	395.8	1371	35.09	3959	1571	4835	37.74	36.25	57.76
**GW0289**	C	345.3	1422	36.68	4091	1466	5177	39.86	37.64	48.60
**GW0290**	C	438.4	1594	45.71	4582	1575	4844	41.92	45.1	51.60
**GW0291**	C	392.1	1272	33.35	3985	1419	5041	32.09	39.99	51.60
**GW0292**	C	438.7	1323	36.93	4302	1503	4858	34.15	37.02	45.69

**GW0293**	C	326.9	1307	30.34	4124	1345	5054	36.99	44.24	51.37
**GW0294**	C	385.2	1381	35.02	4216	1353	5815	33.06	36.74	49.02
**GW0295**	C	420.2	1372	33.7	4203	1499	4600	33.07	34	51.60
**GW0296**	C	351.4	1303	32.35	4096	1381	4782	31.22	37.36	48.82
**GW0297**	C	376.7	1148	31.29	3911	1399	5227	30.45	33.3	48.60
**GW0298**	C	396.4	1407	32.57	4638	1487	5514	35.94	36.74	52.75
**GW0299**	C	319.2	1224	27.87	3871	1341	5058	28.93	30.8	45.29
**GW0300**	C	426.2	1196	29.98	3955	1470	5163	34.2	31.58	49.40
**GW0301**	C	352.4	1351	30.46	3930	1391	5433	30.7	32.78	53.20
**GW0302**	C	454.3	1337	35.24	3910	1403	5919	35.74	31.39	51.00
**GW0303**	C	453.5	1267	33.63	3954	1507	5260	35.07	32.91	46.20
**GW0304**	C	414.2	1236	34.47	3834	1531	5773	36.2	39.72	49.23
**GW0305**	C	338.7	1239	30.14	3648	1421	5541	29.42	30.27	51.54
**GW0306**	C	416.4	1262	34.93	3861	1475	4830	30.28	30.02	57.60
**GW0307**	C	380.9	1317	33.98	3979	1578	5240	34.04	32.55	51.00
**GW0308**	C	394.5	1305	33.41	3971	1428	5619	35.96	33.78	57.45
**GW0309**	C	382.7	1275	35.15	3823	1315	5083	32.5	34.42	57.60
**GW0310**	C	498.1	1290	38.08	3972	1556	5084	36.47	38.48	58.82
**GW0311**	C	422.1	1269	36.23	4014	1465	5373	33.65	33.09	56.86
**GW0312**	C	455.8	1290	30.55	3984	1425	5844	32.67	35.52	49.00
**GW0313**	C	444.6	1331	36.06	3854	1493	5772	36.7	38.86	50.38
**GW0314**	C	335.7	1274	35.42	3738	1334	5123	26.48	31.62	47.65
**GW0315**	C	390	1164	32.41	3647	1400	4751	33.28	31.13	55.00
**GW0316**	C	382.2	1279	34.24	3924	1417	5592	32.48	30.8	51.00
**GW0317**	C	376	1095	31.87	3514	1370	4711	29.58	28.7	48.08
**GW0318**	C	385	1134	31.3	3462	1353	4986	27.6	27.04	51.18

**GW0319**	C	408.2	1331	38.32	4063	1533	5211	35.8	37.83	53.08
**GW0320**	C	338.7	1174	30.62	3777	1397	4799	27.99	28.96	51.37
**GW0321**	C	373.3	1241	37.15	3907	1444	5309	39.81	30.99	58.63
**GW0322**	C	370.2	1324	32.24	3975	1539	5167	35.87	37.29	55.69
**GW0323**	C	448.8	1153	34.63	3619	1398	5315	30.35	29.62	41.96
**GW0324**	C	426.9	1249	31.63	3712	1385	5340	30.78	28.6	45.88
**GW0325**	C	440.2	1143	31.59	3713	1401	5450	31.81	30.9	52.60
**GW0326**	C	405.2	1268	35.05	3960	1452	5467	36.58	34.93	50.58
**GW0328**	C	469.1	1450	37.3	3933	1521	5056	40.84	36.8	56.67
**GW0329**	C	397.4	1155	31.46	3572	1389	4793	35.43	35.44	51.60
**GW0330**	C	334.9	1236	29.32	3778	1513	4849	32.2	30.97	50.78
**GW0331**	C	339.4	1240	30.9	3677	1524	4878	32.99	33.1	57.60
**GW0332**	C	375.4	1165	28.34	3605	1429	5458	31.2	32.63	52.08
**GW0333**	C	554	1319	34.91	4155	1525	5995	34.63	41.31	45.80
**GW0334**	C	299.7	1299	34.35	3914	1658	4754	52.42	43.72	48.65
**GW0335**	C	312.4	1213	32.47	3651	1469	4831	37.01	35.28	51.20
**GW0336**	C	376.1	1337	34.16	4053	1514	5385	40.67	41.84	52.88
**GW0337**	C	368.1	1387	38.49	3989	1458	4693	40.07	43.05	54.90
**GW0338**	C	467	1336	36.79	4489	1666	4468	40.02	42.35	53.40
**GW0339**	C	343.6	1269	32.2	3812	1484	4704	29.02	30.51	56.00
**GW0341**	C	413.5	1189	32.55	3760	1484	4955	35.15	30.69	54.31
**GW0342**	C	Na	Na	Na	Na	Na	Na	44.09	41.45	Na
**GW0343**	C	Na	Na	Na	Na	Na	Na	43.99	52.67	Na
**GW0344**	C	392	1194	34.32	3796	1539	4576	35.37	33.83	52.80
**GW0345**	C	359.1	1263	29.34	3679	1433	4894	30.92	29.68	52.31
**GW0346**	C	326.2	1414	32.62	4193	1467	4824	36.06	33.47	54.80

**GW0347**	C	363.9	1420	33.69	4331	1495	5095	33.63	38.34	56.92
**GW0348**	C	399.1	1194	34.29	3795	1535	5237	36.54	33.15	47.74
**GW0349**	C	447.7	1171	32.51	3846	1474	4844	37.09	36.7	50.00
**GW0350**	C	364.3	1266	34.95	3726	1513	4880	33.47	29.6	53.20
**GW0351**	C	422.4	1250	34.6	4012	1554	5321	31.42	31.53	53.00
**GW0352**	C	449.5	1288	39.54	3977	1544	4741	35.42	38.78	60.20
**GW0355**	C	503	1188	32.95	3969	1502	5425	30.23	31.6	57.65
**GW0356**	C	Na	Na	Na	Na	Na	Na	45.76	50.18	Na
**GW0357**	C	332.4	1344	33.81	4259	1544	4940	32.8	36.42	59.80
**GW0358**	C	370.1	1310	30.25	3870	1531	5305	33.34	35.13	51.96
**GW0359**	C	311.5	1358	30.65	4106	1529	4983	33.27	33.15	52.50
**GW0361**	C	379.2	1212	32.49	3754	1423	5091	31.06	28.03	49.04
**GW0363**	C	Na	Na	Na	Na	Na	Na	26.99	27.25	Na
**GW0366**	C	386.9	1457	32.59	4055	1579	4733	37.49	34.48	56.86
**GW0367**	C	443.8	1496	38.45	4069	1424	4834	33.83	38.46	51.96
**GW0368**	C	349.1	1366	35.4	3826	1491	4522	33.58	32.33	54.80
**GW0369**	C	372	1436	41.04	4117	1602	4608	35.95	39.12	49.61
**GW0370**	C	396.1	1341	36.97	3832	1550	4666	37.71	34.26	53.53
**GW0371**	C	397.4	1624	39.85	4470	1620	4901	40.87	39.52	54.53
**GW0372**	C	372.1	1587	40.13	4386	1691	4965	45.15	42.11	51.63
**GW0373**	C	475.9	1472	37.66	4019	1406	5129	32.41	33.19	55.20
**GW0375**	C	336	1282	35.95	3789	1388	5289	34.77	31.29	61.57
**GW0381**	C	376.1	1288	32.29	4062	1491	5534	37.18	37.44	54.20
**GW0382**	C	302.3	1187	27.45	3681	1385	5167	29.9	30.4	55.60
**GW0383**	C	313.1	1154	29.54	3861	1620	4617	38.39	38.23	50.57
**GW0384**	C	350.7	1222	31.27	3864	1495	4644	36.34	36.21	57.50

**GW0385**	C	Na	1191	29.93	3764	1416	4982	31.05	32.52	Na
**GW0018**	T	552.9	1484	45.02	4475	1449	5386	42.54	47.37	59.20
**GW0105**	T	453.8	1373	43.53	4141	1586	4352	36.26	36.44	54.53
**GW0119**	T	561.2	1374	37.73	4285	1553	4798	32.74	34.1	52.83
**GW0129**	T	508.7	1438	39.13	3873	1420	4774	38.05	33.58	59.60
**GW0141**	T	469.3	1365	38.48	4050	1447	4555	32.32	35.97	52.94
**GW0145**	T	446.5	1503	41.66	4475	1631	4561	39.8	39.91	55.96
**GW0156**	T	590.7	1292	42.71	4061	1512	4612	36.17	42.63	55.31
**GW0160**	T	Na	1503	51.93	4652	1917	4597	48.52	48.74	62.00
**GW0163**	T	533.6	1569	42.81	4718	1959	4144	40.66	47.85	55.58
**GW0168**	T	429.6	1403	37.14	4086	1519	4690	38.66	39.55	62.45
**GW0169**	T	586.5	1420	42.6	4242	1583	4351	39.36	40.4	46.40
**GW0203**	T	391.2	1519	36.95	4662	1657	4552	40.1	41.61	60.80
**GW0209**	T	534.3	1266	39	3822	1411	4645	33.37	33.62	57.60
**GW0216**	T	463	1389	36	3983	1434	4854	31.85	32.98	50.00
**GW0217**	T	396.9	1418	37.61	3893	1420	4514	37.45	35.74	52.55
**GW0219**	T	441.3	1382	35.69	3581	1449	4385	30.8	31.44	58.04
**GW0221**	T	504.6	1368	42.04	3659	1448	4492	34.72	35.4	65.58
**GW0245**	T	433.9	1392	40	4107	1597	4250	36.18	36.08	48.20
**GW0246**	T	481.1	1581	40.85	4427	1586	4677	37.59	40.52	52.88
**GW0247**	T	424	1456	39.93	3833	1600	3995	31.56	30.85	49.22
**GW0248**	T	387.9	1507	39.29	4296	1561	4423	31.06	36.5	53.73
**GW0251**	T	507.8	1706	49.15	4757	1677	4796	39.61	44.77	50.59
**GW0275**	T	441.1	1445	38.6	4066	1530	4702	37.66	39.49	51.40
**GW0281**	T	521.1	1650	38.23	4400	1595	4458	37.66	43.91	47.88
**GW0327**	T	455	1498	36.83	3909	1620	4342	37	35.6	56.33

**GW0354**	T	515.9	1471	41.14	4176	1718	4828	37.64	42.09	55.49
**GW0374**	T	462.7	1468	36.36	4096	1508	4596	33.2	35.08	58.20
**GW0376**	T	455.4	1549	39.82	4215	1548	4470	44.5	30.79	48.43
**GW0377**	T	466.1	1392	36.12	3986	1465	4464	30.72	31.44	53.27
**GW0378**	T	496.3	1471	40.99	4039	1525	4362	35.75	33.63	51.32
**GW0379**	T	509.7	1634	40.38	4357	1529	4219	36.82	42.17	52.94
**GW0380**	T	407.3	1543	38.56	4192	1584	5064	40.61	41.42	48.30
**GW0294a**	C	287.1	Na	Na	Na	Na	Na	34.3	36.18	49.60

Table S6: Expression analysis output for the putative candidate genes underlying nutrient minerals accumulation in wheat grains.

Tissue	TraesCS 2A02 G123400	TraesCS 2B02 G202600	TraesCS 3B02 G006700	TraesCS 3B02 G013300	TraesCS 4B02 G024300	TraesCS 4B02 G293600	TraesCS 4B02 G380200	TraesCS 5A02 G486100	TraesCS 5B02 G012300	TraesCS 5B02 G042900	TraesCS 5B02 G403400	TraesCS 7A02 G169100	TraesCS 7B02 G478200	TraesCS 7D02 G540700	TraesCS 5A02 G542600
Aleurone layer	0.000	2.670	0.014	0.164	1.657	1.971	0.577	2.333	6.512	1.099	2.140	2.824	1.273	1.148	2.405
endosperm	0.000	2.657	0.112	0.015	0.007	1.713	0.082	2.568	5.534	0.527	3.381	2.175	1.661	2.511	0.479
Seed coat	0.021	2.466	0.595	3.143	1.486	3.008	1.345	2.068	5.249	1.051	2.348	2.111	0.178	0.149	1.208
lemma	0.053	3.750	3.120	4.104	0.006	5.442	5.068	2.884	5.581	3.323	2.687	2.087	1.723	1.886	2.825
grain	0.092	2.873	0.828	1.125	0.788	2.090	1.212	2.474	5.728	1.079	2.790	3.490	1.117	1.558	0.906
glumes	0.052	3.716	4.244	3.530	0.013	5.400	5.449	2.832	5.562	3.427	2.727	1.860	2.279	2.820	2.526
rachis	0.053	3.119	4.422	1.956	3.105	3.478	3.951	3.171	7.033	2.867	2.988	3.115	1.423	1.762	1.339
ovary	1.827	4.198	0.086	0.146	3.732	2.016	1.020	2.236	7.500	1.704	3.802	0.270	0.424	0.345	1.782
awns	0.009	3.283	1.866	4.183	0.005	5.292	5.841	2.831	5.487	3.737	2.577	0.529	3.280	3.711	2.840
spike	0.463	4.001	0.924	1.587	1.663	2.339	2.878	1.653	6.159	2.255	3.271	1.086	1.527	1.645	1.615
spikelets	1.403	3.860	5.331	3.715	2.787	4.595	3.780	2.881	6.923	3.183	3.984	4.796	0.658	0.789	3.217
Flag Leaf blade	0.013	3.490	0.037	0.353	0.000	5.609	6.167	2.548	5.504	3.715	2.054	0.813	3.555	3.726	3.306
Leaf ligule	1.406	3.896	4.961	0.782	0.000	5.473	5.569	2.746	5.258	3.202	1.856	2.569	2.391	2.410	4.685
internode	0.147	3.867	1.397	4.168	0.003	4.977	5.076	3.071	6.171	3.257	2.812	4.831	1.658	1.573	2.942
peduncle	0.112	3.611	1.320	4.251	0.003	5.014	4.573	2.676	5.319	3.489	4.208	1.832	1.522	1.817	3.237
stem	1.106	2.794	3.191	4.480	1.986	2.997	2.596	1.961	6.079	2.160	2.838	3.139	1.332	1.054	2.819
shoots	0.148	3.290	1.564	2.813	0.316	4.426	4.870	1.820	6.411	3.396	3.858	2.333	2.890	2.712	2.274
roots	2.378	3.410	0.640	2.372	0.876	2.331	1.569	2.086	6.998	1.498	3.870	5.939	2.592	2.694	0.566
